# Herb-Derived Products: Natural Tools to Delay and Counteract Stem Cell Senescence

**DOI:** 10.1155/2020/8827038

**Published:** 2020-10-08

**Authors:** Provvidenza M. Abruzzo, Silvia Canaider, Valeria Pizzuti, Luca Pampanella, Raffaella Casadei, Federica Facchin, Carlo Ventura

**Affiliations:** ^1^Department of Experimental, Diagnostic and Specialty Medicine (DIMES), University of Bologna, Via Massarenti 9, 40138 Bologna, Italy; ^2^National Laboratory of Molecular Biology and Stem Cell Bioengineering-Eldor Lab, National Institute of Biostructures and Biosystems (NIBB), Innovation Accelerator, CNR, Via Piero Gobetti 101, 40129 Bologna, Italy; ^3^Department for Life Quality Studies (QuVi), University of Bologna, Corso D'Augusto 237, 47921 Rimini, Italy

## Abstract

Cellular senescence plays a very important role in organismal aging increasing with age and in age-related diseases (ARDs). This process involves physiological, structural, biochemical, and molecular changes of cells, leading to a characteristic trait referred to “senescence-associated secretory phenotype (SASP).” In particular, with aging, stem cells (SCs) *in situ* exhibit a diminished capacity of self-renewal and show a decline in their functionality. The identification of interventions able to prevent the accumulation of senescent SCs in the organism or to pretreat cultured multipotent mesenchymal stromal cells (MSCs) prior to employing them for cell therapy is a main purpose of medical research. Many approaches have been investigated and resulted effective to prevent or counteract SC senescence in humans, as well as other animal models. In this work, we have reviewed the chance of using a number of herb-derived products as novel tools in the treatment of cell senescence, highlighting the efficacy of these agents, often still far from being clearly understood.

## 1. Introduction

Aging is a biological process resulting from a continuous interaction between the genome and environmental factors, being characterized by damage accumulation and progressive dysfunction of tissues and organs [1]. In humans, aging carries an increasing risk of developing neurodegenerative disorders, diabetes, osteoarthritis, cancer, and cardiovascular disease, which are commonly referred to as age-related diseases (ARDs) [2], and the outcome of aging is death [3].

It is commonly accepted that cellular senescence plays a very important role in organismal aging and ARDs [4]. In fact, it has been observed that senescent cells accumulate in the tissues and organs of humans and old animals and that the increased production of cytokines by senescent cells and the senescence-associated impairment of regenerative processes can lead to ARDs [5–9].

The role of senescence is complex and depends on the age of the organism [10]. In a young organism, for example, cell senescence exerts beneficial functions. In fact, it is essential in embryonic development (senescent cells are removed by immune cells), in tissue regeneration and as a protection from cancer (senescent cells are not able to proliferate). On the other side, in an old organism, the number of senescent cells increases, and they generate a state of low chronic inflammation, via the so-called senescence-associated secretory phenotype (SASP), that cause microenvironmental changes, which support the aging-related functional decline, the tumor progression [11], and the advancement of multiple ARDs [12–14]. Senescent cells, in fact, increase the production and secretion of proteins, which can act in both an autocrine and a paracrine manner [15–17], to alter their own microenvironment, thus inducing cellular senescence in neighboring cells, remodeling of extracellular matrix, and stimulation of inflammatory processes [18].

Cell senescence refers to physiological, structural, biochemical, and molecular changes that reduce the proliferative potential up to a permanent cessation of cell division, activating the innate immune system in order to remove the senescent cells themselves [3, 18]. In particular, senescence is characterized by altered cellular morphology, cell-cycle arrest associated with increased level of inhibitors (cyclin-dependent kinase inhibitor 1A (p21) and cyclin-dependent kinase inhibitor 2A, multiple tumor suppressor 1 (p16, also known as p16Ink4a)), increased activity of the lysosomal enzyme senescence-associated *β*-galactosidase (SA-*β*-Gal), and permanent DNA damage with activation of the DNA damage response pathway. Moreover, senescent cells exhibit chromosomal instability linked to changes in a chromatin structure due to modified gene expression, as well as higher DNA vulnerability [18–21].

Not only is the cell senescence a property of an organism, but it also occurs in cells cultivated in *vitro* for a long time. For example, multipotent mesenchymal stromal cells (MSCs) are usually employed in regenerative medicine and age in culture when expanded for *in vivo* transplantation [22, 23].

## 2. Multipotent Mesenchymal Stromal Cells and Senescence

SCs exist in most mammalian organs or tissues to preserve tissue homeostasis and participate in tissue maintenance, repair, or regeneration [24–26].

With the aging of an organism, SCs exhibit a diminished capacity of self-renewal and proliferation, which results in an increase of apoptosis or senescence in the SC compartment and in a decline of SC functionality [27]. For example, depletion of neural SCs (NSCs) appears to be responsible for neurogenesis decline with age [28, 29]. Moreover, hair graying is associated to a huge reduction of melanocyte SCs in the hair follicles and to the appearance of mature pigmented melanocytes in the SC niche, both in aged mice and in humans [30].

Among adult SCs, the MSCs, somatic stromal cells with stem-like features [31], are emerging as hopeful candidates for cell-based therapy of numerous diseases [32]. These cells are plastic-adherent cells isolated from bone marrow and other tissues, which express specific surface antigen markers and have multipotent differentiation potential [31]. However, the accurate nomenclature and biological identity of these cells and cell cultures are still the subject of debate. Cell therapy protocols generally require hundreds of million MSCs per treatment, and consequently, MSCs need to be expanded *in vitro* for several weeks before implantation. Notably, donor's clinical history, age, and genetic background strongly influence the length of this expansion period and the quality of the obtained cells. In particular, aged MSCs generally perform less well than their younger counterparts [33–35]. On the other hand, telomeres in old SCs are still longer than those in the other somatic cells of the same tissues [6] as it has been observed in many tissues, including the skin, small intestine, cornea, testis, and brain. These observations suggest that SCs divide at a much slower rate than their proliferative progeny or that they have evolved systems to gain protection against telomere shortening, a main cause of cell senescence [3, 36].

However, SCs do not grow indefinitely, both *in situ* and *ex vivo* (during prolonged expansion), but undergo to a limited number of cell division with progressive arrest of proliferation and occurrence of the senescent phenotype [22, 23].

Senescent MSCs, as the aged somatic cells, exhibit *in vitro* distinctive morphological features, such as an enlarged, flattened, and irregular morphology [26, 35, 37]. They also show a high SA-*β*-Gal activity (index of lysosomes malfunction) used as a marker of senescence both *in vitro* and *in vivo* [8], associated with the modulation of p16 expression [38, 39]. Another hallmark of senescent MSCs is that cells sustain metabolism, but gradually lose mitosis reactivity and DNA synthesis activity, resulting in their arrest in G1 phase without entering S phase [40]. The cell cycle arrest is mediated by the overexpression of inhibitory proteins such as tumor protein p53 (p53), p21, and p16 and by the downregulation of proteins stimulating cell replication, like cyclins, the Fos protooncogene AP-1 transcription factor subunit (c-Fos), and the proliferating cell nuclear antigen (pCNA) [41]. In particular, p16 prevents cells from escaping G1 phase by binding cyclin-dependent kinase 4 (CDK4) to inhibit the assembly of the cyclin D-CDK complex [42]. At the same time, p21 prevents cells from entering S phase, in which DNA synthesis occurs. As a result of the overexpression of p21, cells arrest in G1 phase, and cell senescence is induced. Senescent MSCs are also characterized by the presence of DNA damage and heterochromatin foci in which it is possible to identify some markers for DNA damage as the phospho-histone H2A.X gamma (p-*γ*H2AX) and the telomere associated foci [43]. Moreover, mitochondrial dysfunction and the consequent oxidative metabolism imbalance contribute to develop the MSC senescence phenotype [44, 45]. In fact, superoxide dismutase (SOD), an important antioxidant enzyme, decreases its activity, while the production of reactive oxygen species (ROS) increases: the latter degrades polyunsaturated lipids, thus forming malondialdehyde (MDA), whose levels continuously increase with cell age [46]. Finally, experimental data strongly suggest that also *ex vivo* cultured MSCs show the SASP-like phenotype, typical of senescent somatic cells in the organism [26, 47]. SASP involves the secretion of hundreds of molecules, of which interleukin- (IL-) 1*α*/*β*, IL-6, IL-8, the transforming growth factor- (TGF-) *β*, and the tumor necrosis factor- (TNF-) *α* are the most characterized [47–49]. In particular, IL-1*α* is an upstream master regulator of the SASP, while IL-1*β* and TGF-*β* mediate senescence spread, with the downstream products IL-6 and IL-8 reinforcing senescence in an autocrine fashion [50, 51]. SASP is mostly induced by the nuclear factor kappa B subunit (NF-*κ*B), the main transcription factor of the immune system [52, 53]. There is strong evidence that also sirtuins (SIRTs) can modulate cell senescence, extending lifespan/health-span, in different animal models [54]: in particular, SIRT6 regulates telomere-independent cell senescence by inhibiting NF-*κ*B [55].

A deep investigation and understanding of the functional consequences of SC senescence is therefore a crucial endeavor in both *in vivo* and *in vitro* studies: *in vivo* in order to delay and counteract aging processes in the organism and *in vitro* in order to obtain large amounts of MSCs retaining a high clinical therapeutic potential, based upon their characteristic paracrine effects, differentiation potential, immunomodulatory activity, and migration ability [23, 26].

## 3. Cell Senescence Induction

There is compelling evidence showing that certain features of cell senescence and mechanisms of its induction can differ depending on the analyzed cells and on the senescence type [56]. This concerns, for example, a different inclination of cells to respond to stress [57], some metabolic differences between stress-induced and replicative senescence [58–61], or differences in SASP components [62].

In fact, cells can undergo senescence as a result of different conditions. These include a progressive telomere erosion linked to a replicative impairment (*i.e*., telomere-dependent, replicative senescence) [63] or to the formation of irreparable DNA lesions ensuing into a persistent DNA damage response, which keeps the cells indefinitely alive nevertheless arresting their proliferation and making them susceptible for subsequent apoptosis (*i.e*., telomere-independent, premature senescence) [18, 57]. The stress-induced premature senescence (SIPS) can occur in response to some internal stimuli (increased ROS production, oncogene overexpression, endoplasmic reticulum-stress, DNA damage, and chromatin structure dysfunction) or external stimuli (chemical and physical factors) [59]. For instance, the overexpression of activated oncogenes [52] conducts to the formation of senescence-associated heterochromatin foci in relation to the upregulation of p16Ink4a [18, 64].

In different cell types, premature senescence can be induced by multiple extrinsic chemical and physical agents [18]. Researchers have taken advantage of multiple extrinsic chemical and physical agents to induce artificially the aging of the organism or the senescence of tissue-derived SCs, in the attempt to uncover the underlying mechanisms and develop novel antisenescence strategies in specific animal or cellular models.

Among the stressors used in this field, there are hydrogen peroxide (H_2_O_2_), D-galactose (D-gal), ferric ammonium citrate (FAC), lead acetate, tert-butyl hydrogen peroxide (t-BHP), and lithium chloride (LiCl). Moreover, oxidative stress, defined as an imbalance between the productions of free radicals/ROS and antioxidants [65], contributes significantly to DNA damage and cellular senescence [66]. For this reason, H_2_O_2_ treatment is commonly used as a model for assessing cellular susceptibility to oxidative stress. Although MSCs appear to handle efficiently oxidative stress, they undergo premature senescence *in vitro* when exposed to H_2_O_2_ [67, 68]. To this end, H_2_O_2_ was found to hamper (stem) cell viability in a dose- and time exposure-dependent manner [69, 70], provoking DNA breaks [71], together with a raise in the number of SA-*β*-Gal-positive cells [70], the altered expression of senescent marker genes, as well as *p53* and *p21*, and the increase of apoptosis, with a decline of prosurvival gene expression [69].

D-gal is also used to induce premature senescence *in vitro* interfering with the balance between ROS and antioxidant enzymes. D-gal is a reducing sugar that is metabolized at a normal concentration in the body. However, its excess leads to the accumulation of aldohexose and H_2_O_2_ under the action of galactose oxidase and promotes the generation of ROS and the superoxide anion, damaging the function of macromolecules and cells [72]. *In vivo*, mice continuously exposed to D-gal show the decline in antioxidant defense enzymes, such as SOD [73, 74].

Moreover, an excess of iron can lead to an oxidative stress condition with toxic effects on SCs. Iron is an essential micronutrient, and it is required as a cofactor of enzymes involved in fundamental cellular processes such as DNA synthesis, oxygen transport, and cellular respiration. Although continued iron deficiency could cause cell death, on the other hand, free iron excess could be toxic. Iron overload is a disease characterized by excessive iron deposition in tissues, damaging vital organs including the heart, liver, and kidney [75–77]. Recently, iron accumulation has been shown to impair the bone marrow microenvironment and suppress the proliferation and differentiation of human bone marrow-derived mesenchymal stromal cells (BMSCs), thus leading to lower bone mineral density, and postmenopausal, age-linked osteoporosis [78]. It was shown that FAC is able to induce a condition of iron overload that markedly reduces the viability and proliferation of BMSCs, coaxing them into apoptosis and senescence [79].

Lead acetate represents another cell stressor used in research to induce senescence. Lead (plumbum) is one of the most ubiquitous environmental toxins, and the exposure to it produces deleterious outcomes to organism functions, including those of the nervous, reproductive, hematopoietic, and renal systems [80–82]. Lead acetate is able to induce the senescence transition of hematopoietic stem into progenitor cells (HSCs/HPCs) increasing the transcriptional expression of *p53* and *p21*, enhancing SA-*β*-Gal activity and promoting the production of proinflammatory cytokines IL-6 and TNF-*α*, indicating a SASP development [83]. Moreover, lead exposure led to a significant reduction in white blood cells, red blood cells and hemoglobin, and perturbed cell quiescence of HSCs, indicated by the increased cell proliferation of LSK (Lin−sca-1+c-kit+) populations [83].

Some senescence inducers interfere directly with specific molecular pathways. For example, the t-BHP is able to modulate the SIRT6-NF-*κ*B signaling pathway, stimulating the expression of SIRT6 that inhibits NF-*κ*B and induces the senescent phenotype [55]. Otherwise, LiCl is able to regulate the Wnt/*β*-catenin signaling pathway [84]. It is known that a treatment of NSCs with LiCl resulted in a decrease of cell viability and proliferation, which corresponds to a significant decline in the percentage of cells in G1 and S phases of the cell cycle. Moreover, in the same conditions, the percentage of SA-*β*-Gal staining was enhanced indicating that the *in vitro* senescence model of NSCs was successfully established by adding LiCl [85].

To induce SIPS [64], researchers used also a physical insult, the x-ray exposure [47, 52]. It has been reported that ionizing radiation causes tissue acute damage and long-term bone marrow injury, including DNA damage, which primarily contributes to the senescence and the reduction of self-renewal and pluripotency of HSCs [86, 87]. In fact, the most sensitive cells to the acute effects of radiation are the most replicative cells, such as lymphohematopoietic elements [88].

## 4. Antiaging Approaches and Aim of Our Literature Review

Increasing positive living conditions (as food availability and medical treatment) have been contributing to extend life expectancy in developed countries, raising the proportion of elderly individuals in the population [89]. At the same time, several approaches resulted in the rejuvenation of aged SCs in which intrinsic changes observed at genomic, epigenomic, and proteomic levels are potentially reversible [3]. The demonstration that SC clearance is sufficient to delay ARD development and to extend lifespan in mice has prompted intense research into the pathways leading to SC build-up, in order to develop new tailored therapeutic strategies to prevent SC accumulation, contain their secretory activity, achieve their selective elimination, and reduce possible off-target effects [90].

Therefore, a number of natural and synthetic compounds have been investigated for their antisenescence and antiaging potential in cellular and animal models as well as in humans [91, 92]. Among the several tested molecules with antiaging and SC-protection properties, there are for example resveratrol and melatonin. Recently, Zhou and colleagues [93] showed that resveratrol treatment prevents H_2_O_2_-induced premature senescence of MSCs while Shuai and collaborators [94] demonstrated that melatonin improves MSC therapy by preserving stemness, thus suggesting melatonin as a promising candidate to optimize MSC expansion *in vitro*. At the same time, in BMSCs recovered from old mice or in a senescence-accelerated mouse-prone 6 (SAMP-6) model, a treatment with resveratrol inhibited MSC senescence in a dose-dependent manner and promoted the ability to osteogenic differentiation [93, 95].

In general, antiaging approaches can be divided into two main categories: a delay-aging approach and a treat-aging approach. The delay-aging approach consists in using agents capable of preventing aging and the associated decline of the organism functions. The antiaging treatment can be administered to the organism or to its cells before or together with the proaging agent (chemical or physical) that is used to induce organism aging and/or cell senescence *in vivo* and *in vitro.*

On the other hand, the treat-aging approach consists in using agents capable of reducing age-associated changes in organisms and cells. In this case, the antiaging treatment is administered to old organisms or to their senescent cells after the exposure to proaging agents (chemical or physical).

**Figure 1:**
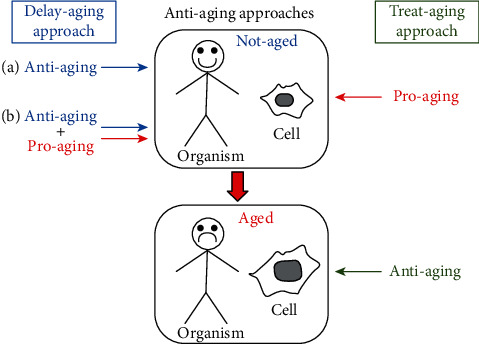
Antiaging approaches: delay-aging approach (on the left side)—the antiaging treatment is administered to the organism or to its cells before (a) or together (b) with the proaging agent; treat-aging approach (on the right side)—the antiaging treatment is administered to the old organism or to its senescent cells (physiologically aged or induced with a proaging agent).

Since in medical research, we noticed that antiaging and antisenescence approaches (on organisms and on cells, respectively) were often based on the use of the same treatment; here, we decided, for simplicity, to use only the word “aging” for aging itself and for senescence in Figure 1.

The aim of our research was to review the many scientific studies published about the role played by herb-derived products in preventing, counteracting, or reversing human and animal SC senescence *in vivo* and/or *in vitro*.

## 5. Literature Search and Article Selection

We performed a systematic literature search to identify articles related to treatments used to slow down senescence in SCs derived from humans and other animals.

Since many studies with different approaches have been conducted on this topic, we started with the definition of suitable criteria to search for and choose the articles discussed herein.

The first search was conducted on PubMed up to March 2020, using the terms antisenescence or anti-senescence AND “stem cells”; antiaging or anti-aging AND “stem cells”; antiageing or anti-ageing AND “stem cells.” All searches were then filtered for the following terms: “plant” OR “herb” OR “herbal.”

In a second search, the terms senescence OR aging OR ageing AND “stem cells” were used. All searches were then filtered for the following terms: “plant” OR “herb” OR “herbal.”

Results obtained were then analyzed in order to identify articles of interest and to exclude repeated data. In particular, we did not include in our study descriptive articles related only to molecular pathways involved in the modulation of SC senescence and researches referred to the use of MSCs to counteract senescence of other cells/tissues or the aging of the whole organism.

**Figure 2:**
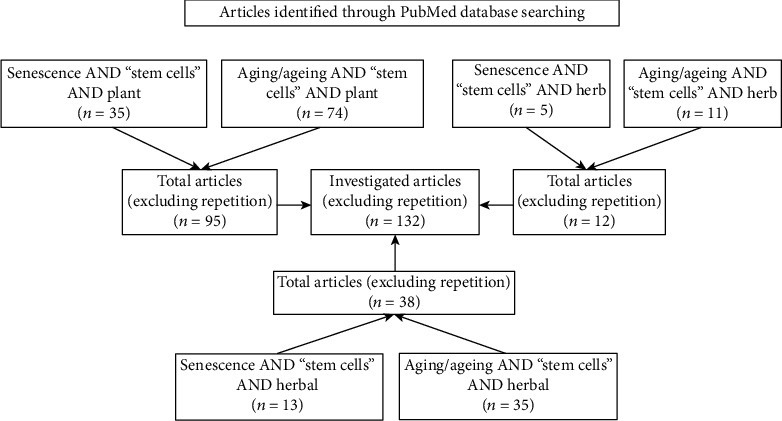
Flow diagram of PubMed data searching.

All results selected in the first research (23 articles, using detailed criteria of exclusion) were also found in the second search. In Figure 2, we represent the flow diagram of our second search in PubMed database and the numbers of useful articles retrieved, then selected on the basis of the declared criteria.

The search was also conducted with the use of the following Mesh terms: “Plants, Medicinal”[Mesh], “Herbals as Topic”[Mesh], “Herbal Medicine”[Mesh] and “Drugs, Chinese Herbal”[Mesh] instead of “plant” or “herb” terms.

Finally, in order to deep the analysis of identified plants/herbs, we performed another PubMed search using plant/herb name AND “stem cells,” then filtered for “aging” OR “ageing” OR “senescence” terms. With these further researches, it was possible to enrich the amount of useful results to study the properties of specific antiaging agents.

## 6. Analysis of Antiaging and Antisenescence Protocols

Analyzing the literature, we have identified diversified research protocols to study the effects of herb-derived products or their known active compounds on SC biology.

Researchers can administer antiaging treatment to old animals or to aging animal models, in order to study the effects on the whole animal (*i.e.* changes in serum molecules, organ size, animal weight, and cognitive abilities), or to senescent SCs directly in their niche (*in situ*) or to *ex vivo* cultured tissue-derived SCs, such as MSCs, short-term HSCs, and NSCs.

**Figure 3:**
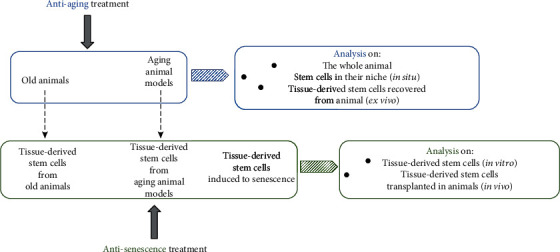
Antiaging and antisenescence research protocols. In antiaging protocols, the animals receive the treatment and then the effects on the whole organism or on its cells are analyzed. In antisenescence protocols, senescent cells receive the treatment and the effects are directly analyzed in culture or after cell transplantation.

In other studies, an antisenescence treatment has been applied to senescent tissue-derived SCs of old animals or of aging-animal models or to cells induced to senescence with physical or chemical approaches. In the last protocol, effects of antisenescence treatment were analyzed in *ex vivo* cultured tissue-derived SCs or in an animal (*in vivo*) after transplantation of the treated senescent cells (Figure 3).

## 7. Herb-Derived Products with an Antiaging and Antisenescent Role

Several studies have shown that single herbs, herb extracts, and specific herbal compounds play a role in regulating SC proliferation and differentiation [96], as well as influencing the senescence of SCs. Several herb-derived products have also been suggested to promote organism health and longevity [97, 98].

We report below herbs in which unknown or known active compound(s) counteract the aging process and SC senescence. From our literature, emerged effects on SCs *in situ* or on *ex vivo* cultured tissue-derived SCs both in animal models and in human. We decided to resume the principal data of our analysis in four tables (Table 1–3 and Table S1).

### 7.1. Effects of Herb-Derived Products on Animal Senescent Cells

In this paragraph, are reported herb-derived products that show antiaging properties and/or antisenescent role on SCs *in situ* and/or on *ex vivo* cultured tissue-derived SCs isolated from animals or recovered after animal treatment.

#### 7.1.1. Siraitia grosuenorii and Rehmannia glutinosa

The fruits of *Siraitia grosuenorii* (Swingle) C. Jeffrey ex A.M. Lu & Zhi Y. Zhang, cultivated in China, are used as a natural sweetening agent and it has been reported to have beneficial effects in the diabetic subjects for its antihyperglycemic role [99]. Many reports have also shown that it has antitumoral and antioxidant properties [100, 101].

Bai and colleagues [102] fed mice with 200 mg/day of *S. grosuenorii* for 10 months, and this treatment led to a slower aging process with a slight increase in the animal lifespan. It is known that the hematopoietic system of the aged mice shows T- and B-lymphoid cell impairment and an increase of the number of myeloid cells. Therefore, aged HSCs showed reduced self-renewal activity and reduced hematopoiesis reconstructive ability [103]. Interestingly, in the study of Bai et al., the delay and prevention of the aging process elicited by *S. grosuenorii* resulted from the enhanced function of HSCs *in vitro* (recovered after animal treatment), responsible for resumption in blood cell production. In fact, *S. grosuenorii* contributes to maintain the quiescence of HSCs, which is essential to avoid the premature depletion of SCs and to conserve the HSC pool throughout life [104]. This herb was also able to reduce ROS levels with the downregulation of the cell senescence-associated gene expression, *p21*, *p53*, and *p16*. It is known that the loss of HSC quiescence is frequently correlated with an increased presence of cellular ROS [105]. Moreover, it was demonstrated that in HSCs, *S. grosuenorii* leads to a reduction of the number of SA-*β*-Gal-positive cells and to an increase of telomere length, highlighting the reemerging of characteristic traits of younger cells [102].

*Rehmannia glutinosa* (Gaertn.) DC. shows similar effects on HSCs, compared to those of *S. grosuenorii*. In the past few years, pharmacological studies on *R. glutinosa* have focused mainly on their broad actions on the blood and on the endocrine, cardiovascular, and nervous systems. In particular, this herb-derived product is used to treat diabetic disorders, being able to enhance the bone metabolism in osteoporosis and to inhibit liver inflammation and fibrosis [106, 107]. In addition, *R. glutinosa* has antifatigue, antidepressant, and neuroprotective properties [108, 109].

In a further study by Bai and colleagues, mice fed with 200 mg/day of *R. glutinosa* for 10 months showed characteristics of a slowed aging process, including a decrease in cell senescence and an increase in survival. HSCs harvested from treated animals showed *in vitro* the maintenance of HSC quiescence with an upregulation of the *cyclin-dependent kinase inhibitor 2C* (*p18*) gene expression and an increase in the number of cells in the G0 phase of the cell cycle. A decreased number of SA-*β*-Gal-positive cells and a reduction of ROS levels with a downregulation of cellular senescence-associated proteins p53 and p16 were also evident. Thus, *R. glutinosa* was shown to possess strong immunoenhancement activity, with an important role in the prevention of cell and animal aging [103].

In a study of Zhou et al. [110], *R. glutinosa* was also used together with “Chinese Angelica” (*Angelica sinensis* (Oliv.) Diels) in a formulation named “HuangDiSan” to investigate its role in association with Sanjiao acupuncture on NSCs. It is known that in SAMP-8 mice Sanjiao acupuncture can promote proliferation, migration, and differentiation of endogenous NSCs in main distribution areas, as the subventricular and subgranular zones (SVZ and SGZ) of the hippocampus [111], improving learning and memory impairment, as well as behavior in the dementia-model. In particular, SAMP-8 mice subjected to a 15-day combined treatment with acupuncture and HuangDiSan (intragastric administration 0.2 mL/day) prior to exogenous NSC transplantation, followed by additional 15 days postsurgery in the presence of the same combinatorial treatment, showed a significant improvement of learning ability and an increase in synaptophysin mRNA/protein levels in the hippocampus. Moreover, combined treatment promoted the proliferation, migration, and differentiation of exogenous NSCs more than acupuncture alone. This observation indicates that the HuangDiSan formulation can affect the NSC microenvironment enhancing the positive impact of the transplantation of exogenous NSCs, through a reduction in the presence of their senescent phenotype [110].

The role of *R. glutinosa* in nervous system function was investigated also by Park and coworkers [112] in association with other herb-derived products. These authors investigated the beneficial effects exerted by Polygonum Multiflorum Thunberg Complex Composition-12 (PMC-12), a mixture of four medicinal herbs (*Reynoutria multiflora* (Thunb.) Moldenke also known as *Polygonum multiflorum* Thunb, *Polygala tenuifolia* Willd, *R. glutinosa*, and *Acorus gramineus* Aiton), on hippocampal neurogenesis, learning, and memory in mice. *Reynoutria multiflora* is a popular traditional herbal medicine in East Asia, and its extracts have been found to protect from oxidative stress-associated neurodegenerative diseases [113] and to have potential therapeutic effects against aging, hyperlipidemia, Alzheimer's disease (AD), Parkinson's disease, inflammation, and cancer [114]. *Polygala tenuifolia* and *A. gramineus* and their major active compounds have been reported to improve memory and cognitive function [115, 116]. Park et al.'s study showed that a dose of 500 mg/kg/day for 2 weeks reduced latency times in treated C57BL/6 mice in association to an increased neural plasticity and hippocampal neurogenesis. In fact, after treatment in mice, hippocampal NSC proliferation and the survival of newly generated cells were increased, together with a raise in the levels of a number of markers for neural plasticity and neurogenesis, including brain-derived neurotrophic factor (BDNF), the cAMP response element-binding protein (p-CREB), and synaptophysin [112].

#### 7.1.2. Acori tatarinowii, Fuzhisan, and Yokukansan

Acori Tatarinowii Rhizoma (AT), the root of *Acorus calamus var. angustatus* Besser also known as *Acorus tatarinowii* Schott, is a traditional herbal medicine widely used in China for the treatment of brain disorders, such as senile dementia, dysmnesia, and stroke. Several pharmacological studies have revealed that AT possesses neuroprotective effects [117, 118] and improves learning and memory in aged, dysmnesia murines [119, 120] and ischemic rats [121].

In the nervous system, the aging process is related to oxidative stress, microglial activation, and proinflammatory factors, which are known to reduce cell proliferation and to limit neuroplasticity. These factors may lead the transition from normal aging to more severe cognitive dysfunction associated with neurodegenerative diseases [122]. In particular, aberrant neural progenitor cell (NPC) proliferation and self-renewal have been linked to age-related neurodegeneration and neurodegenerative disorders including AD. In a study by Mao and coworkers, it was demonstrated that 100 *μ*L of AT extract (equal to 200 mg AT per 20 g of mouse body weight) and its active compounds, asarones, promoted NPC proliferation, and neurogenesis in the hippocampus of aged mice and retarded deficits of NPC proliferation and neurogenesis in transgenic AD model mice when treated by gavage once per day for 28 days. AT and its fractions also enhanced in a dose-dependent manner the proliferation of NPCs cultured *in vitro* for 24 hours (h), activating extracellular signal regulated kinase (ERK) but not the serine/threonine kinase 1 (Akt), two critical kinase cascades for neurogenesis. At the same time, AT treatment did not affect NPC lineage commitment [123].

The role of AT on the nervous system was also investigated in a Chinese herbal mixture, the Fuzhisan (FZS), containing Ginseng root (*Panax ginseng* C.A. Mey.), Baical skullcap root (*Scutellaria baicalensis* Georgi), rhizome of A*corus calamus L.* (*A. talarinowi*), and Radix Glycyrrhizae (*Glycyrrhiza uralensis* Fisch.) in the proportions of 4 : 6 : 3 : 1, respectively [124]. Yang and colleagues submitted aged SAMP-8 mice to an intragastric administration of FZS (2.4 and 4.8 g/kg), once daily for 30 consecutive days. Results showed that FZS improved impaired cognitive ability, by enhancing hippocampal neurogenesis with proliferation of NPCs and prolonged survival of the newborn cells in the hippocampal dentate gyrus (DG). To elicit these effects, FZs significantly increased the number of BrdU- (bromodeoxyuridine-) positive cells in a dose-dependent manner in the SGZ and in the SVZ of the lateral ventricles, indicating a tendency to stimulate NSC proliferation. Finally, FZS increased the survival rate of new-born cells (*i.e*., the percentage of BrdU-positive cells at 30 days after BrdU injection relative to that at 24 h) and induced neuronal differentiation in the hippocampal DG of SAMP-8 mice [124].

Yokukansan (YKS, yi-gan san in Chinese) represents another mixture of crude drugs (*Atractylodis Lanceae Rhizoma*, *Poria*, *Cnidii Rhizoma*, *Uncariae Uncis* cum *Ramulu*s, *Angelicae Radix*, and *Bupleuri Radix*) that was widely used, with very few side effects, in several clinical situations for treating symptoms associated with age-related neurodegenerative disorders, such as behavioral and psychological symptoms of dementia [125–127] and sleep disturbance in patients with dementia [128]. YKS improves learning disturbance and aggression in a rat model of AD [129, 130] and ameliorates beta-amyloid-induced neurotoxicity *in vitro* [131]. These findings have prompted Tanaka and colleagues to investigate whether YKS may be effective for age-related structural degeneration of cognition-responsible brain regions, such as the prefrontal cortex and the hippocampus. They treated old rats (twenty-one months old) with powdered YKS for 3 months, demonstrating that this drug decreased the age-related increase in aggrecan expression (a major molecule of chondroitin sulfate proteoglycans) throughout the prefrontal cortex and in the hippocampus, while increasing the proliferation of NSCs, identified by BrdU incorporation. Moreover, it was shown that the migration of NSCs from regenerating regions was enhanced [132].

#### 7.1.3. Ginkgo biloba

*Ginkgo biloba* L. grows in China, Japan, and Korea. Phytochemical studies have established a typical composition of the extract from this plant: 24% of phytoestrogens (kaempferol, kercetin, and ishorhamnetin), 6% of terpenoids (ginkgolides and bilobilides), and less than 5 ppm of ginkgolic acid [133].

*Ginkgo biloba* extract (GBE) is a traditional herbal product extracted from the leaves of the ginkgo tree. GBE is known for its specific actions in improving blood flow, protective actions against damage by free radicals and anti-inflammatory effects [134–137]. Moreover, in mouse BMSCs, it inhibits adipocyte differentiation and enhances osteogenic differentiation, as demonstrated by the increase of calcium deposition and alkaline phosphatase activity, a marker for osteogenic differentiation, and by upregulation of the expression of the osteogenic genes including the *bone morphogenetic protein 2* (*BMP-2*), *RUNX family transcription factor 2* (*RUNX2*), and *collagen type I alpha 1 chain* (*Col1a1*). At the same time, GBE is able to decrease the mRNA expression of the adipogenic *peroxisome proliferator-activated receptor gamma* (*PPARγ*) and *adipocyte protein 2* (*ap2*) genes in a dose-dependent manner, with no effect on the proliferation of BMSCs cultured *in vitro* [133].

In a study of Osman and coworkers, the treatment of elderly mice (2 years old) with a dose of 100 mg/kg GBE once daily for 28 days decreased the age-related decline of adult hippocampal neurogenesis. In particular, adult hippocampal SCs of DG increased, as pointed out by the higher number of Ki-67 (marker of proliferation) positive cells and by the increased proportion of neuronal marker-expressing cells in the SGZ. Moreover, an antiapoptotic effect of GBE treatment was inferred from a decreased number of caspase3-positive cells in DG hippocampal sections, as compared to nontreated controls [138].

GBE exhibited also antioxidative and antiapoptotic effects when it was applied to treat neural damage and disorders. In the Organ of Corti, oxidative stress can result in a hearing derangement, a condition for which NSCs hold great therapeutic potential. In a study by Wang and coworkers, a treatment with 50 mg/mL of GBE promoted cell survival and proliferation of NSCs isolated from mouse cochlea, increasing the size of neurospheres in formation. In addition, GBE treatment stimulated NSC differentiation to neurons (with a specific increase in the number of cells positive to MAP2, a neuron-specific cytoskeletal protein) and enhanced the performance of mature neural networks with an increased frequency of calcium oscillation and neurite outgrowth [139].

Wang and colleagues also investigated GBE effects on mouse cochlear NSCs that were committed to an oxidative stress model using 0.25 *μ*M H_2_O_2_. GBE cotreatment at the concentration of 50 mg/mL for 24 h was found to attenuate H_2_O_2_ effects. In fact, GBE increased NSC viability and prevented mitochondrial depolarization and subsequent cell apoptosis, by antagonizing the intrinsic mitochondrial apoptotic pathway. Moreover, the addition of GBE to the H_2_O_2_-treated NSCs attenuated ROS production and fully restored glutathione (GSH) level and SOD activity to almost the same levels as the respective controls. These findings support the potential therapeutic value of GBE in preventing oxidative stress-related hearing loss, resulting from the age-related imbalance between ROS generation and antioxidant defenses [140].

A specific antisenescence role of GBE was also investigated in human SCs by Dong and colleagues. These authors treated endothelial progenitor cells (EPCs) with different concentrations of GBE (10, 25, and 50 mg/L) and found that GBE increased cellular proliferation and prevented the naturally occurring cellular senescence, enhancing telomerase activity through the activation of the phosphatidylinositol 3-kinase (PI3k)/Akt signaling pathway [141].

#### 7.1.4. NT-020

A nutraceutical combination of blueberry, green tea extract, carnosine, and vitamin D3 (a proprietary formulation known as NT-020) was examined in aged Fisher rats to investigate its effects on neurogenesis and animal performance [122].

It was known that NT-020 is able to stimulate the proliferation of human BMSCs, bone marrow-derived CD34+, and progenitor cells from peripheral blood (CD133+) *in vitro* [142]. Moreover, NT-020 supplementation protects male Sprague-Dawley rats against ischemic stroke, decreasing by 75% mean glial scarring at the infarction area, increasing the proliferation of SCs in the SVZ of hippocampus, and the migration of SCs to the area of injury [143].

In the study by Acosta and coworkers, the analysis of animals treated with NT-020 135 mg/kg per day for a period of 4 weeks at the end of treatment revealed an evident improvement in cognitive function with optimization of spatial memory performance. To obtain these effects, NT-020 promoted health, proliferation, and maintenance of neurons in the aged animals and exerted anti-inflammatory actions in the aged SC niche. In fact, after 1 month of NT-020 dietary supplementation in aged rats, NSC proliferation and neurogenesis were increased in the SGZ of the hippocampal DG, with a significant decrease in the major histocompatibility complex class II expression, to indicate a decline in brain inflammation [122].

#### 7.1.5. Elaeocarpus sylvestris

*Elaeocarpus sylvestris* (Lour.) Poir. is a genus of tropical and subtropical evergreen trees and shrubs. Its region of distribution includes the subtropical zone, from Cheju in South Korea to Southern China, Okinawa, Kyushu (Japan), and Taiwan. *Elaeocarpus sylvestris* extract contains 1,2,3,4,6-penta-O-galloyl-beta-D-glucose, which has been reported to suppress tumor growth via inhibition of angiogenesis, to exert an antiproliferative effect on the human hepatocellular carcinoma (cell line SK-HEP-1) [144], and to inhibit oxidative DNA cleavage by scavenging the superoxide and generating hydroxyl radicals [145].

Park and colleagues (2008) studied the potential of *E. sylvestris* to protect mice from radiation injury by single whole-body irradiation *in vivo*. 25 mg/kg was injected intraperitoneally in the animals at day 1 before, at the time of and 3, 6, and 9 days after irradiation. *Elaeocarpus sylvestris* extract significantly improved the rate and duration of animal survival and enhanced the regeneration of HSCs in the spleen, as revealed by the increase in the counts of endogenous colony-forming units (CFU) in extract-treated animals [88].

In Table 1, the main studies (above described) about herb-derived products with antisenescence role on animal senescent cells are resumed.

### 7.2. Effects of Herb-Derived Products on Human Senescent Cells

Here, we report herb-derived products that show antiaging properties and antisenescent role in human *ex vivo* cultured tissue-derived SCs.

#### 7.2.1. Andrographis paniculata

*Andrographis paniculata* (Burm.f.) Nees, also known as the “King of Bitters,” belongs to the family Acanthaceae. It is a traditional herbal medicine widely used in Asian countries. The pharmacological properties of *A. paniculata* have been mainly attributed to andrographolide, the major active compound which has been found to have anticancer, antidiabetic, antibacterial, anti-inflammatory, and antioxidative properties [146]. Moreover, the antiaging activity of *A. paniculata* extract has been demonstrated by You and coworkers [147]; in their studies, the treatment of human epidermal SCs (EpSCs) with *A. paniculata* (1-10-30 *μ*g/mL) promoted cellular proliferation by the enhancement of the Integrin *β*1 and the vascular endothelial growth factor (VEGF) expression, both involved in SC maintenance. In addition, the same authors demonstrated that *A. paniculata* stimulated the Integrin *β*1 expression in human skin explants [147]. Furthermore, the VEGF released in the conditioned medium of EpSCs treated with *A. paniculata* increased collagen synthesis in human dermal fibroblasts, thus limiting the aging process. Finally, in a clinical study, the treatment with a formulation containing *A. paniculata* improved skin quality in healthy female volunteers confirming its antiaging properties [147].

#### 7.2.2. Cirsium setidens

*Cirsium setidens* (Dunn) Nakai is a wild perennial plant, which is found in Korea. It was used in the treatment of hemostasis, hematemesis, hematuria, and hypertension [148] and showed anticancer, antioxidative, antiadipogenic, and hepatoprotective properties [149–151], attributable to its bioactive compounds, such as hispidulin 7-O-neohesperidoside, pectolinarin, luteolin, and apigenin. The antioxidant activity of *C. setidens* was also investigated on human adipose-derived stem cells (ASCs), which were pretreated with *C. setidens* (100 *μ*g/mL) and then exposed to a prooxidant agent (H_2_0_2_, 200 *μ*M) [69]. Results demonstrated that *C. setidens* increased MSC viability and suppressed H_2_O_2_-induced ROS production through the inhibition of the activation of the mitogen-activated protein kinases (MAPKs). Moreover, *C. setidens* inhibited H_2_O_2_-induced apoptosis modulating the ATM serine/threonine kinase (ATM)/p53 signaling pathway which, in turn, upregulated and downregulated the expression of antiapoptotic and proapoptotic proteins, respectively [69].

#### 7.2.3. Dhanwantharam kashaya

*Dhanwantharam kashaya* (DK) is a commercial synthetic herbal formulation containing about 40 herbs; it is widely used in Ayurvedic medicine to promote growth in children, to enhance nerve regeneration, to treat nervous system diseases, and to prevent postpartum complications. As reported in several studies, DK has antioxidant activity [152, 153]. Warrier and colleagues observed that the treatment of the human Wharton's jelly MSCs (WJMSCs) with DK (10 *μ*g/mL) enhanced cell proliferation and viability, improving MSC quality without affecting cell stemness features. Furthermore, the reduction in the number of positive SA-*β*-Gal cells and the downregulation of *p21* expression suggested that DK was able to delay senescence during WJMSC long-term culture [154].

#### 7.2.4. Du-Huo-Ji-Sheng-Tang

Du-Huo-Ji-Sheng-Tang (DHJST) is a Chinese herbal medicine composed by 15 herbs: *Angelica pubescens* Maxim, *Taxillus chinensis* (DC.) Danser, *Eucommia ulmoides* Oliv., *Cyathula officinalis* K.C.Kuan, *Asarum sieboldii* Miq., *Gentiana crassicaulis* Duthie ex Burkill, *Wolfiporia cocos* (Schw.) Ryvarden, *Cinnamomum cassia* (L.) J. Presl, *Saposhnikovia divaricata* (Turcz.) Schischk., *Ligusticum striatum* DC, *P. ginseng*, *G. uralensis*, *A. sinensis*, *Paeonia lactiflora* Pall., and *R. glutinosa*. It is used in the treatment of osteoarthritis, rheumatoid arthritis, and osteoporosis [155–157]. To investigate the biological mechanism that might explain the effects of DHJST on preventing osteoporosis, Wang and colleagues treated human MSCs with DHJST (3 and 6 *μ*g/mL) or with *L. striatum* also known as *Ligustium chuanxiong* (1 and 3 *μ*g/mL), a major active component of DHJST, and evaluated their ability to promote osteogenic activity. The results showed that both DHJST and *L. chuanxiong* increased the osteogenic differentiation in the absence of a specific osteogenic induction medium. In addition, both DHJST and *L. chuanxiong* reduced cellular senescence during MSC long-term culture [158].

#### 7.2.5. Myrtle Extract

Myrtle (*Myrtus communis* L.) is a medicinal plant growing in the Mediterranean area. It is known that berries, branches, leaves, and fruits possess a broad range of pharmacological properties including antimicrobial, anti-inflammatory, and antioxidant activity [159]. It was demonstrated that the exhausted berries of myrtle (bioproducts), resulting from myrtle liquor production, contained a high content of bioactive molecules, such as polyunsaturated fatty acids and phenolic compounds with high antioxidant activity [160]. Cruciani and colleagues found that the pretreatment of ASCs (12-24 and 48 h) with myrtle bioproducts (0.5 mg/mL) counteracted oxidative stress, inflammation, and senescence, induced by H_2_0_2_ exposure (100 *μ*M for 1 h). Moreover, the authors demonstrated that in this model myrtle bioproducts reduced H_2_0_2_-induced senescence through the increase of SIRT-1 expression and enhanced ASC regenerative potential by upregulating the expression of pluripotency-related genes (*octamer-binding transcription factor 4* (*Oct4*), *Nanog homeobox* (*Nanog*), and *SRY-box transcription factor 2* (*Sox2*)) [161]. Recently, the same authors investigated the molecular mechanisms underlying the antisenescence effect of myrtle bioproducts in H_2_O_2_-senescent ASCs. Data demonstrated that myrtle bioproducts decreased the expression of cell cycle regulators (p16Ink4a, ARF tumor suppressor (p19ARF), p21, and p53) and increased the expression of the telomerase reverse transcriptase (TERT) and MYC protooncogene, bHLH transcription factor (c-Myc) in H_2_O_2_-senescent ASCs [162].

#### 7.2.6. Tianshengyuan-1

*Tianshengyuan-1* (TSY-1) is a Chinese herbal medicine obtained from the extraction of multiple Chinese herbs. In China, it is used to treat bone marrow deficiency diseases such as aplastic anemia (AA) and myelodysplastic syndrome, which are characterized by abnormalities in telomerase activity. It was demonstrated that TSY-1 enhanced hematopoiesis in the immune-mediated AA animal model modulating the telomerase activity of hematopoietic cells [163]. In order to elucidate the molecular mechanism exerted by TSY-1 on telomerase activity, in a recent study, Yu and colleagues treated the human leukemia cells HL60, the healthy donor-derived human PBMCs, and the blood CD34+ HSCs with TSY-1 (31.2 and 62.5 *μ*g/mL) for 24 h. They found that TSY-1 increased telomerase activity in normal blood mononuclear and in CD34+ HSCs; on the contrary, in the HL60 cell line, the authors observed an inhibition of telomerase activity. The opposite effects of TSY-1 on telomerase activity in normal and cancer cells correlated with the different number of positive SA-*β*-Gal staining cells and may be related to a different epigenetic modulation of the TERT promoter which, in turn, resulted in a different expression of TERT gene and protein [164].

#### 7.2.7. Tinospora cordifolia and Withania somnifera

*Tinospora cordifolia* (Thunb.) Miers and *Withania somnifera* (L.) Dunal are commonly used plants in the traditional Ayurvedic medicine [165, 166]. *Tinospora cordifolia* (Guduchi/Giloy) belongs to the Menispermaceae family and grows in the south of India and in China. *Tinospora cordifolia* exhibits several pharmacological proprieties, including antioxidant, anti-inflammatory, antimicrobial, anticancer, and immunomodulatory activities. Moreover, it was reported that *T. cordifolia* exhibited cardioprotective and neuroprotective activities as well as antiaging properties [167–169]. The wide spectrum of *T. cordifolia* properties was attributed to the presence of different bioactive components, such as alkaloids, sesquiterpenoids, diterpenoids, phenolics, steroids, aliphatic compounds, and polysaccharide [165]. *Withania somnifera* (Ashwagandha), also known as Indian winter cherry and Indian ginseng, belongs to the family of Solanaceae. *Withania somnifera* root extracts contain a complex mixture of several compounds, including alkaloids and lactones, which exhibit a broad range of biological activities and exert beneficial effects in the treatment of different conditions, such as anxiety, depression, cognitive impairments, stress, and cancer [166]. In MSCs, the antisenescence effect of *T. cordifolia* and *W. somnifera* was investigated for the first time by Sanap and coworkers. The authors demonstrated that both *T. cordifolia* leaf extract (10 *μ*g/mL) and *W. somnifera* root extract (5 *μ*g/mL) promoted cell proliferation, inhibited apoptosis, and delayed senescence in human WJMSCs, suggesting that both extracts improved cell quality [170].

#### 7.2.8. Undaria pinnatifida

*Undaria pinnatifida* Harvey (Suringar) is a brown, edible seaweed used in the traditional Chinese medicine or as a functional food source. The biological activities of *U. pinnatifida* and its main components, fucoxanthin and fucoidan, were recently summarized in a review published by Zhao and colleagues [171]; *U. pinnatifida* showed anti-inflammatory, antioxidant, anticancer, and antiobesity properties. The antioxidant and antisenescent effects of *U. pinnatifida* extract were tested in human BMSCs [172]. The preincubation of BMSCs with *U. pinnatifida* extract (5 *μ*g/mL) for 24 h, increased cell viability, reduced cellular damage, and decreased ROS production in H_2_0_2_-treated cells (1 mM H_2_O_2_ for 1 h) through the recovery of the antioxidant enzyme expression (SOD-1, SOD-2, and catalase). Moreover, the treatment with *U. pinnatifida* extract attenuated the cellular replicative senescence, modulating the ROS production induced by cell expansion, and maintained the differentiation potential in long-term cultured BMSCs [172].

In Table 2, the main studies (above described) about herb-derived products with an antisenescence role on human *ex vivo* cultured tissue-derived SCs are resumed.

### 7.3. Effects of Known Herbal Active Compounds on Animal and Human Senescent Cells

Here, we report known herbal active compounds with antiaging properties and/or antisenescent role in animal and/or human senescent cells.

#### 7.3.1. Ginsenosides Rg1 and Rd

Ginseng, the root of *P. ginseng* (Araliaceae), has been used to enhance stamina and the body's capacity to address fatigue and physical stress for thousands of years in Chinese medical science, and it is now commonly used around the world [87]. The beneficial effects of ginseng and its constituents in terms of its anticancer and immunomodulatory effects have also been reported [173]. The modern medical practice has proved that ginseng has an “invigorating qi and promoting blood” effect on a variety of causes of bone marrow dysfunction as well as anemia [174].

It has also been demonstrated that ginsenoside Rg1, one of major 25 constituents derived from ginseng, has various pharmacological actions including antiaging, antioxidant, and immunomodulatory effects [175–177]. In fact, this saponin, besides regulating cell cycle and protein expression [87], inhibits telomere DNA damage and improves the activity of telomerase to delay aging [74]. Moreover, Rg1 enhances EPC angiogenic potency [178] and antagonizes the HSC senescence, regulating the blood cell production and improving the immune function with consequent antitumor effects.

In particular, in mice treated with radiation to induce senescence, Rg1 enhances the resistance to the aging process of HSCs by inhibiting the expression of p16Ink4a and p21, both at gene and protein levels, and by enhancing the production of hematopoietic cytokines, such as cell factor (SCF) and granulocyte-macrophage colony-stimulating factor (GM-CSF) [87, 179, 180].

Similarly, Rg1 has antiaging effects on MSCs directly treated with D-gal or isolated from animal after D-gal and Rg1 administration. After treatment of rats with D-gal and Rg1, the recovered BMSCs showed enhanced antioxidant and anti-inflammatory properties and a strong ability to resist to hematopoietic microenvironment senescence. In fact, cells showed a reduced percentage of SA-*β*-Gal-positive cells and a reduced number of apoptotic bodies, as well as a slowdown of ROS levels and MDA activity linked to an increased SOD activity. Moreover, it was shown that there was a reduced expression of inflammatory markers (IL-6, IL-2, TNF-*α*, and IL-1*β*) and senescence associated proteins (p16, p21, p53), as well as an increased S phase cell percentage and enhanced SCF and GM-CSF expression [174, 181]. Even HSCs recovered by treated D-gal mice showed a decrease in the properties typical of the cell senescent phenotype when Rg1 was administered to animals. In HSCs, Rg1 induced an enhanced CFU-Mix ability, an improvement of oxidative stress indices, analyzed through the assessment of ROS, total antioxidant (T-AOC), SOD, GSH-px, and MDA, and a downregulation of advanced glycation end products and of the H2A.X (r-H2A.X)/8-hydroxy-2′-deoxyguanosine (8-OHdG) indicators of DNA damage [74]. HSCs showed a similar behavior also when they were recovered after treatment of rats with lead acetate and then Rg1 [83].

When BMSCs were cotreated *in vitro* with D-gal and Rg1 [174] or when Sca-1+ HSCs, exposed to the senescence inducer t-BHP, were pre-/posttreated with Rg1 [55, 182, 183], the same type of effects was evident, but the cellular responses were more accentuated in Rg1-pretreated than in Rg1-posttreated cells. Rg1 prevented the decrease in colony number, reversed the enhancement of p16Ink4a and p21 expression, and upregulated the expression of cyclinD1, decreasing cells in G1 phase. Additionally, Rg1 increased the mRNA and protein expression of SIRT6, leading to a downregulation of the NF-*κ*B pathway [55, 183].

Various studies revealed that ginsenoside can also prevent cognitive impairment marked by the memory loss and the decrease of spatial learning in mice. Ginsenoside Rd is able to stimulate active neurogenesis in adult hippocampus, a brain region closely related to animals' learning and memory ability, increasing the numbers of BrdU+ and DCX+ cells in the hippocampal DG, while unaffecting NSC differentiation [184]. At the same time, this compound maintains neurogenesis after lead-induced neural injury [185, 186], and ginseng total saponins improve the neurorestoration in rats after traumatic brain injury [187].

In particular, Rg1 is able to attenuate changes in the hippocampus, including the cognitive capacity, the expression of senescence-related markers, and hippocampal neurogenesis in old mice/rats or following D-gal [177] treatment. It was shown that Rg1 increased the hippocampal cell proliferation, enhanced the activity of the antioxidant enzymes, decreased the levels of IL-1*β*, IL-6, and TNF-*α*, increased the DNA telomere lengths, and downregulated the mRNA expression of cellular senescence associated genes *p53*, *p21*, and p19 [177].

Compounding the antisenescence action of Rg1, this ginsenoside was also able to antagonize the senescent process elicited in NSCs by LiCl, which produces brain senescence by activating the Wnt/*β*-catenin signaling pathway [85]. In particular, Rg1 increased the number of proliferative NSCs, while significantly reducing the percentage of senile neurospheres and the expression of the nuclear catenin, T-cell factor (Tcf), lymphoid enhancer factor (Lef), glycogen synthase kinase 3 beta (p-Gsk-3*β*), and c-Myc [85].

#### 7.3.2. Angelica Polysaccharide and Astragalus Membranaceus Polysaccharide

*Angelica sinensis* (Oliv.) Diels (dong quai) has been for centuries a renowned remedy in traditional Chinese medicine for the treatment of hematologic and gynecological diseases. Angelica Sinensis Polysaccharide (ASP) is a major ingredient in *A. sinensis* with significant bioactivities, including antioxidant, antitumor, antiaging, antihepatotoxic, immunomodulatory, and neuroprotective effects [188].

ASP can antagonize D-gal injury to organs such as the liver, kidney, and spleen [189–191] and can protect HSC/HPCs against X-ray-irradiation-induced aging, by inhibiting oxidative stress damage [192] and increasing telomerase activity [193]. In fact, in C57BL/6J mice, X-ray irradiation significantly increased the cell ratio of HSC G1 stage, the rate of SA-*β*-Gal-positive cells, and the expression of p53 protein and reduced the length of telomere and the vitality of telomerase [193]. On the other hand, intragastric administration of ASP during X-ray irradiation significantly decreased the production of ROS and remarkably increased the capability of T-AOC in HSCs. In addition, ASP downregulated the expression of *p16* mRNA and increased the capacity of CFU in HSCs, when compared with the aging group without ASP treatment [192]. Mu and colleagues employed the D-gal-induced aging mouse model to further explore the antiaging role of ASP in HSC/HPCs *in vivo*. Mice were treated with D-gal (120 mg/kg·bw)/day for 42 days, or they were administered with intraperitoneal ASP from day 8^th^ of D-gal injection. As a consequence of ASP treatment, in HSC/HPCs, the levels of ROS, 8-OHdG, and 4-HNE declined, with a concomitant reduction in the levels of 𝛾-H2A.X, a marker of DNA damage (double strand breaks). Therefore, ASP decreased the expression of effectors p16Ink4a-RB and p19Arf-p21Cip1/Waf in senescent pathways and inhibited the excessive activation of Wnt/*β*-catenin signaling [194].

By investigating the effects of ASP, different studies concluded that ASP has the role of protecting NSCs against aging [195, 196]. Nestin-green fluorescent protein (GFP) transgenic mouse is a model constructed to explore the antiaging process and be able to detect the number, distribution, differentiation, and migration pathways of NSCs in the brain tissue using fluorescence-labeled nestin. In a study of Cheng and colleagues, nestin-GFP-transgenic mice were treated with D-gal (200 mg/kg)/day for 42 days and in the last 28 days was added a treatment with 140 mg/kg/day of ASP. Animals showed a slowdown of cognitive impairment and a decrease in the percentage of senescent neurospheres in the hippocampus. Even NSCs treated with 100 *μ*g/mL of ASP for 24 h after D-gal senescence induction showed a reduction in the expression of the senescent phenotype. In fact, owing to ASP treatment, cell proliferation increased in a dose-dependent manner, the levels of both MDA and ROS were reduced, and conversely, the activity of SOD and T-AOC resulted to be increased. Finally, inflammatory cytokines (IL-1*β*, IL-6, and TNF-*α*) were also reduced and the cellular senescence-associated genes *p53* and *p21* were downregulated [196].

*Astragalus propinquus* Schischkin (commonly known as *Astragalus membranaceus* (Fisch.) Bunge) is one of the most commonly used antiaging herbs in traditional Chinese medicine (Huang Qi), and it has been widely used to treat a variety of diseases such as diabetes and myocardial infarction with positive clinical outcomes [197]. In China, it is also marketed as a life-extending tonic for humans [98]. The significant components of *A. membranaceus* are polysaccharides, flavonoids, and saponins. The components of *A. membranaceus* have been shown to increase telomerase activity and mediate antioxidant, anti-inflammatory, immunoregulatory, anticancer, hypolipidemic, antihyperglycemic, hepatoprotective, expectorant, and diuretic effects [98]. Astragalus Polysaccharide (APS) is a major active ingredient of *A. membranaceus*. Recent studies further indicated the diversity of the potential effects of APS on improving microcirculatory disturbances, including antioxidation, inhibition of apoptosis, and amelioration of injury to target organs such as the kidney [198, 199].

Yang and colleagues demonstrated that the treatment of mice BMSCs with APS (dose from 30 to 100 *μ*g/mL) impeded mitochondrial ROS accumulation and remarkably inhibited apoptosis, senescence, and the reduction of both proliferation and pluripotency caused by FAC-induced iron overload. Iron accumulation has been shown to impair the bone marrow microenvironment, thus leading to lower bone mineral density and bone loss in mice [200], in addition to human postmenopausal osteoporosis [78]. In the study of Yang et al., the treatment of BMSCs with APS counteracted the multifaceted detrimental effects induced by iron overload, including the decrease in proliferation and viability, the reduction of *Nanog*, *Sox2*, and *Oct4* expression, and the increase of intracellular and mitochondrial ROS levels. APS also attenuated iron overload-induced apoptosis (partly prevented the increase of the BCL2-associated X protein, Bax, and the reduction of the BCL2 apoptosis regulator (BCL-2)) and reduced the percentage of SA-*β*-Gal-positive cells [79].

Moreover, *A. radix* combined with *A. sinensis radix* was able to improve HSC dynamics in a senescence model induced by using t-BHP. The combinatorial use of both drugs inhibited HSC senescence promoted HSC proliferation, as well as cell cycle remodeling by upregulating the expression of cell cycle positive regulators (Cyclin D1) and downregulating the expression of cell cycle-negative regulators (p53 and p21). On the whole, these actions coaxed HSCs to reenter the proliferation phase from a stationary phase [201].

#### 7.3.3. Allicin

Garlic (*Allium sativum* L.) is widely consumed, and mounting studies have identified that garlic shows ameliorating roles in multiple diseases, such as cardiovascular disease and cancer. It has been reported that those protective effects are associated with allicin, which is the product of interactions between alliinase and alliin and is emitted by cutting and crushing garlic cloves. Allicin is considered to represent a potential therapeutic agent for osteoarthritis, with several favourable outcomes, resulting from antioxidant, immunomodulatory, anti-inflammatory, antidiabetic, and antigenotoxic properties [202–205]. Allicin also elicited ameliorative effects in *Pasteurella multocida*-infected rabbits, nephroprotective effects on cisplatin-induced toxicity, and a beneficial response towards doxorubicin-induced cardiotoxicity [206]. In addition, allicin afforded an antioxidant role on *Nile tilapia* and antiaging effects in H_2_O_2_-stressed human umbilical vein endothelial cells [207].

Intragastric administration of allicin substantially ameliorated lead acetate-induced HSC senescent phenotypes and animal aging. Rats cotreated with allicin significantly ameliorated SASP features by reducing IL-6 and TNF-*α* levels in the peripheral blood and by reversing the imbalance in the differential population of myeloid and lymphoid cells in the bone marrow, in addition to improving colony-forming ability of LSK population containing HSCs. Moreover, in HSCs, allicin attenuated the increased cellular ROS production and DNA damage (reduced expression of *γ*-H2AX) and alleviated cell senescence by upregulating the pyruvate kinase PKM2 (a kinase involved in mediating intracellular ROS levels) [208].

#### 7.3.4. Icariin, Curcumin, and Tetramethylpyrazine

Icariin represents the major active compound found in *Herba Epimedii* (also called Ying-Yang-Huo), which is a famous Chinese herbal medicine that is widely used to treat some ARDs, as cardiovascular diseases and osteoporosis in oriental countries, and it is able to improve sexual and neurological functions, prolonging animal lifespan. Different studies have suggested that icariin may improve learning and memory deficits in animal models. Wu and colleagues for example demonstrated that the treatment of old Sprague-Dawley rats with a dose of 0.02 g icariin/kg body weight/day for 3 months induced a beneficial effect on cognitive function in aging rats and the activation of quiescent NSCs in the hippocampus with an increase in cell number and proliferation [209].

Curcumin (diferuloylmethane) is a naturally phenolic yellow chemical, isolated from the rhizomes of the plant *Curcuma longa* L. (turmeric). Because of its ability to scavenge free radicals and to inhibit inflammation, curcumin has been investigated for cancer chemoprevention and tumor growth suppression [210, 211]. In 2008, Kim and coworkers explored the effects of curcumin on NSCs *in vitro* and *in vivo*. At low doses (0.1 and 0.5 *μ*M), curcumin increased the proliferation of primary embryonic cortical NSCs through the activation of the MAPK (ERK and p38 kinases) pathway, and it increased the number of newly generated cells in the DG in adult mice, enhancing hippocampal neurogenesis [212].

Recent findings suggest the possibility that curcumin can reduce oxidative damage and cognitive deficits associated with aging [213]. Studies in animal models have suggested that curcumin may be beneficial in neurodegenerative conditions such as AD [214, 215] and focal cerebral ischemia [216], associated to oxidative damage and cognitive deficits.

In 2017, Yang and colleagues decided to encapsulate curcumin in both silk fibroin films (silk/cur films) and nanoparticles (silk/cur NPs), and their antiaging effects were compared with free curcumin in solution, with the aim to elucidate the mechanism of antiaging of silk-associated curcumin and to better serve biomedical applications. Rat BMSC senescence was retarded in all free curcumin, silk/cur films, and silk/cur NPs samples, with the silk-cur being superior to the curcumin alone. The interaction between the surface exposed curcumin with SCs significantly inhibited cell senescence, as indicated by the downregulation of *p53* and *p16* genes and by reduction of SA-*β*-Gal staining [217].

Antisenescent effects of curcumin were showed also when rat ASCs were treated for 48 h with a dose of 1 and 5 *μ*M of this component. Curcumin increased cell proliferation, significantly decreased the number of senescent cells, and enhanced the expression of *tert* gene [218].

The main bioactive component extracted from the Chinese herb Chuanxiong (*L. striatum* also known as *Ligusticum wallichii*), was represented by tetramethylpyrazine (TMP), a biologically active alkaloid, with a neuroprotective role [219], in addition to an anti-inflammatory [220] and antiaging effect *in vivo*. Therefore, it is widely used to reduce ischemic brain injury [221] and acute spinal cord injury [222]. Nowadays, a large number of researches have demonstrated that TMP serves promoting roles in proliferation [223], in differentiation of cells cultured *in vitro* [224], and in protection of cells from oxidative damage [225].

In view of the wide range of neurotrophic and neuroprotective effects, TMP was used for 4 days at the concentration of 30–50 mg/L on BMSCs in order to study its antisenescent effects. TMP significantly increased cell viability, delayed BMSC senescence by suppressing NF-*κ*B signaling and enhancing the self-renewal ability of BMSCs, and their potential for neuronal differentiation. In particular, TMP reduced SA-*β*-Gal staining by suppressing NF-*κ*B signaling whose activation accelerates tissue and cellular senescence [226]. A series of proinflammatory factors, such as TNF-*α* and IL-1*β*, have been indicated to activate the NF-*κ*B signal pathway [227], and intriguingly, treatment with TMP reduced the expression of these factors.

Additional evidence in support of an antisenescence action of TMP can be inferred by the finding that TMP treatment (40–50 mg/L) can facilitate the neuronal differentiation of BMSCs, as it is highlighted by the presentation of a neuronal morphology, the expression of neuronal markers, such as microtubule-associated protein 2 (MAP-2) and neuron-specific enolase, and the enhanced expression of *neurogenin 1* (*Ngn1*), *neuronal differentiation 1* (*NeuroD*), and *mammalian achaete–scute homolog 1* (*Mash1*) genes [228].

#### 7.3.5. Morin, Vanillin, and Zingerone

Morin (3,5,7,2′,4′-pentahydroxyflavone) is a flavonoid found in *Maclura pomifera* (Raf.) C.K. Schneid. (Osage orange) and *Maclura tinctoria* (L.) D. Don ex Steud. (old fustic) and in the leaves of *Psidium guajava* L. (common guava). It displays anti-inflammatory, antioxidant, and anticancer as well as neuroprotective effects against neurodegenerative diseases [229, 230]. Lee and colleagues demonstrated a protective effect of morin against senescence induced by UVB radiation in human keratinocyte SCs (KSCs). In fact, the treatment with morin (10-20-100 *μ*M) in UVB-irradiated KSCs (30 mJ/cm^2^) resulted in an increase in cell viability together with a decrease in DNA damage and in the number of cells positive to the *β*-Gal-staining. In addition, morin treatment led to a significant reduction in the synthesis of some inflammatory cytokines in UVB-damaged KSCs, confirming its anti-inflammatory activity [231].

Vanillin (4-hydroxy-3-methoxybenzaldehyde) is a natural compound derived from vanilla beans of the *Vanilla planifolia* Jacks. ex Andrews. Vanillin possesses antioxidant, anti-inflammatory, and anticancer properties [232, 233]. Similar to morin, vanillin displays protective effects in human UVB-damaged KSCs. Lee and coworkers demonstrated that vanillin enhanced cell viability and decreased inflammatory cytokine production, senescence, and DNA damage in KSCs irradiated with UVB (30 mJ/cm^2^) [234].

The authors demonstrated that the vanillin- and morin-mediated protective effects in human UVB-damaged KSCs involved the inhibition of ATM/p53/MAPK pathway [231, 234].

Zingerone is one of the nonvolatile pungent compounds which is found in Ginger, the rhizome of *Zingiber officinale* Roscoe. Ginger and its compounds are used to treat or prevent age-related neurological disorders such as neurodegenerative diseases, dementia, and epilepsy [235]. Zingerone has a broad range of pharmacological properties including antioxidant, anti-inflammatory, anticancer, antihyperlipidemic, and antimicrobial activities [236]. In a recent study, Lee and colleagues showed that also zingerone promoted cell viability, reduced inflammation, and attenuated senescence in human UVB-irradiated KSCs. The Zingerone-mediated protection against UVB damage is due to the upregulation of survival-related gene expression (*TERT*, *histone deacetylase 1* (*HDAC1*), and *DNA methyltransferase* (*DNMT1*)) and of proliferation-related gene expression (*sPCNA* and *VEGF*) and to the downregulation in the expression of cell cycle arrest-related genes (*p21*). The mechanism of action by which zingerone exerted its protective effects against UVB irradiation involved the inhibition of p38 and p42/44 MAPK signaling pathways [237].

#### 7.3.6. Quercetin

Quercetin (3,3′,4′,5,7-pentahydroxyflavone) is a common flavonoid which is found in numerous plants, fruits, and vegetables, such as onions and apples. It inhibits PI3K, other kinases, and serpins [238]. Like other flavonoids, quercetin shows antioxidant activity and exhibits neuro, cardiovascular, and cancer protection activities [239, 240]. It is also known that quercetin acts as a cellular senescence modulator, inducing senescence in cancer cell lines and delaying this process in normal cell lines [241].

After a 50 mg/kg/day administration of quercetin by oral gavage to old mice, a reduced presence of SA-*β*-Gal-positive cells and a decreased expression of *p16* mRNA were observed in the fat [242]. At the same time, in the BMSCs obtained from progeroid Ercc1+/D mice, treated with 100 *μ*M quercetin for 48 h, the number of senescent cells was reduced [242]. Moreover, the quercetin-3-O-glucuronide, a quercetin glucuronate primarily detected in the plasma and in the brain, showed to have positive effects on neurogenesis, suggesting its therapeutic potential in neurodegenerative diseases [243].

In a recent study, Geng and coworkers used human Werner syndrome (WS) MSCs, obtained from direct differentiation of WRN^−/−^ hESCs, as a premature senescent SC model, to screen a library of genoprotective active molecules. Among these, quercetin (100 nmol/L) was able to counteract cellular senescence, to enhance cellular self-renewal, and to promote osteogenic and chondrogenic differentiation capabilities as well as to restore the heterochromatin architecture in late-passage WS MSCs. The genome-wide RNA sequencing and Gene Ontology analysis revealed that quercetin modulated genes involved in different biological processes related to cell cycle, cell division, chromosome segregation, and cell proliferation. The attenuation of cellular senescence was confirmed in another premature senescent SC model, Hutchinson-Gilford progeria syndrome hMSCs, as well as in physiological-aging hMSCs [244]. On the contrary, in another study published by Grezella et al.'s group, quercetin (100 *μ*M) did not exert any senolytic effects on replicative senescent hMSCs. Moreover, quercetin did not enhance culture expansion, telomere length, or epigenetic rejuvenation [245]. The hormetic effect of quercetin might explain the discrepancy in the results obtained by Geng and Grezella's groups. In fact, it was demonstrated that quercetin showed different dose-dependent effects on cells [246].

#### 7.3.7. Sesamin

Sesamin is a major lignan constituent of sesame (*Sesamum indicum* L.) and possesses various health-promoting effects. Many *in vitro* and *in vivo* studies have demonstrated its biological effects, including antioxidant [247, 248], anticarcinogenic [249, 250], and antihypertensive effects [251]. Previous studies have demonstrated that sesamin extends the lifespan of *Drosophila (Sophophora) melanogaster* (Meigen, 1830) and *Caenorhabditis elegans* (Maupas, 1900) [252, 253] and corrects oxidative damage-related tissue dysfunction in mammals.

Le and colleagues demonstrated several antiaging effects of sesamin on several aging-related phenotypes in the muscle, brain, and midgut using the Drosophila senescence-accelerated models (Sod1n1 mutant and Sod1-depleted flies) by immunostaining experiments. Sesamin (0.35 and 2 mg/mL) administration extended the lifespan of the fly models. In fact, sesamin feeding suppressed the age-dependent impairment of locomotor activity with reduction of accumulation of damaged proteins and inhibited the accumulation of ROS in animal bodies. Moreover, sesamin partially suppressed the loss of dopaminergic neurons in adult brains displaying ROS accumulation and suppressed the accumulation of DNA damage and hyperproliferation in intestinal SCs. Four antioxidative genes and two DNA repair genes were simultaneously upregulated in sesamin-fed animals [254].

Main studies about animal and human SCs are resumed in Table S1 and Table 3, respectively.

## 8. Conclusions

Cellular senescence represents a main cause of organismal aging. Senescent cells, characterized by the loss of proliferating ability and a SASP phenotype, increase with age and in ARDs, influencing the surrounding cells and the microenvironment. The removal of senescent cells delays the onset of aging and reduces changes related to ARDs [11].

The possibility of employing senescent MSCs, obtained from elderly individuals or induced to senescence as an *in vitro* screening system, can be important to study and discover compounds able to attenuate this process. It is now increasingly becoming evident that the natural herbal environment offers a number of multivariate agents that may hold promise for rethinking the whole senescence process as a programmed pattern rather than, or in addition to, an adaptive process dominated by the stochastic accumulation of detrimental events, ensuing in a loss of cellular identity and function. This view becomes even more intriguing when placed within the context of SC biology. In actual fact, the chance of using natural chemistry to transform SC senescence into a reprogrammable phenomenon provides the clue for merging a rejuvenation process with the enhancement of our inherent ability for self-healing. Herbal compounds are now subjected to advanced screening and can become part of interventions either amenable to prevent the accumulation of senescent SCs *in vivo*, thus reducing their negative impact on organism, or to pretreat MSCs prior to employing them for cell therapy [18].

In this work, we have tried to identify through literature review the main herbs or their known active compounds that resulted to be useful to prevent or counteract SC senescence in humans and other animal models. We have also highlighted the “one/few component(s)-multiple-target” features displayed by these agents, still far from being clearly characterized.

## Figures and Tables

**Table 1:** Herb-derived products with an antisenescence role on animal senescent cells.

Herb name	Studied cells	Animal source	Treatment dose	Time exposure	Senescence inducers (dose of exposure)	Treatment (pre-, post-, and coinducer)	Effects on recovered cells	Effects on animals or cells in animals after treatment	Ref. number
*Acorus tatarinowii*	Hippocampus-resident NPCs after animal treatment NPCs recovered by the hippocampus	C57BL/6 mice (8-month-old AD and 18-23-month-old mice) C57BL/6 mice (6- to 8-weeks old)	100 *μ*L/day (animals) 1 mg/mL (cells)	28 days (animals) 24 hours (cells)	Absent	/	Enhanced cell proliferation in a dose-dependent manner; unaffected NPC lineage commitment	Enhanced neurogenesis and retarded deficits of NPC proliferation both in aged and in AD model mice	[123]

*Elaeocarpus sylvestris*	Spleen-resident HSCs after animal treatment	C57BL/6 mice (8–11 months old)	25 mg/kg/day (animals)	Unknown	X-ray (1.5 Gy/min)	Pre-, post-, and cotreatment	/	Enhanced mouse survival; recovered spleen size; inhibited immune suppression; enhanced cell regeneration and proliferation in the spleen	[88]

*Fuzhisan*	Brain-resident NPCs after animal treatment	SAMP-8 mice	Up to 4.8 g/kg/day (animals)	30 days (animals)	Absent	/		Stimulated neurogenesis in SGZ and SVZ; increased proliferation of NPCs in the SGZ; increased long-term survival of newborn cells in hippocampal DG; stimulated neuronal differentiation in DG	[124]

*Ginkgo biloba*	NSCs recovered by cochlea	Early postnatal BALB/c mice	50 mg/L (cells)	12-24-36 hours (cells)	H_2_O_2_ (0.25 *μ*M)	Cotreatment	Promoted cell viability; attenuated oxidative stress; prevented mitochondrial depolarization and apoptosis; enhanced the spontaneous calcium oscillations in NSC-differentiated neural networks	/	[140]

*Ginkgo biloba*	Hippocampus-resident NSCs after animal treatment	Mice (24 months old)	100 mg/kg/day (animals)	28 days (animals)	Absent	/	/	Decreased number of apoptotic cells in the hippocampal DG; increased number of SC pool and cell proliferation in the SGZ of the hippocampal DG; increased cell differentiation and maturation of newborn neurons and neuroblasts in the hippocampus	[138]

*HuangDiSan*	Exogenous NSCs transplanted in the hippocampus after 15 days of animal treatment	SAMP8 mice	0.2 mL/day (animals)	30 days, with a day off (animals)	Absent	/	/	Improved learning, memory impairment, and behavioral function; promoted proliferation, migration, and differentiation of transplanted NSCs; improved synaptophysin mRNA and protein levels in the hippocampus	[110]

*NT-020*	Hippocampus-resident NSCs after animal treatment	Fischer rats (20 months old)	135 mg/kg/day (animals)	4 weeks (animals)	Absent	/	/	Improved cognitive function with optimization of spatial memory performance; increased proliferation and neurogenesis in SGZ of the hippocampal DG; decreased MHC class II-expressing cells	[122]

*PMC-12*	Hippocampus-resident NPCs after animal treatment	C57BL/6 mice (5 weeks old)	100 or 500 mg/kg/day (animals)	2 weeks (animals)	Absent	/	/	Reduced latency times; increased cell proliferation; increased survival of newly generated cells in the DG; increased levels of BDNF, p-CREB, and synaptophysin associated with neural plasticity and hippocampal neurogenesis	[112]

*Rehmannia glutinosa*	HSCs recovered from animal after treatment	C57BL/6J mice (10 months old)	200 mg/day (animals)	10 months (animals)	Absent	/	Decreased cell numbers; increased cell proliferation capacity; maintained cell quiescence with upregulation of p18; decreased number of SA-*β*-Gal+cells; decreased ROS levels with downregulation of p53 and p16	Maintained body weight; increased animal lifespan	[103]

*Siraitia grosuenorii*	HSCs recovered from animal after treatment	C57BL/6J mice (10 months old)	200 mg/day (animals)	10 months (animals)	Absent	/	Increased telomere length; increased cell proliferation capacity; maintained cell quiescence; decreased number of SA-*β*-Gal+cells; decreased ROS levels with downregulation of p21, p53, and p16	Decreased senescence; increased slightly the body mass; slightly increased animal lifespan	[102]

*Yokukansan*	Brain-resident NSCs after animal treatment	Rats (21 months old)	Concentration of 3% (*w*/*w*) with food pellets (animals)	3 months (animals)	Absent	/	/	Decreased the age-related increase in aggrecan expression throughout the prefrontal cortex and in the hippocampus; increased cell proliferation in the prefrontal cortex and hippocampus; increased migration of NSCs/NPCs	[132]

Herb-derived products are shown in alphabetical order. AD: Alzheimer's disease; BDNF: brain-derived neurotrophic factor; DG: dentate gyrus; HSCs: hematopoietic stem cells; MHC: major histocompatibility complex; NPCs: neural progenitor cells; NSCs: neural stem cells; p16: cyclin-dependent kinase inhibitor 2A, multiple tumor suppressor 1; p18: cyclin-dependent kinase inhibitor 2C; p21: cyclin-dependent kinase inhibitor 1A; p53: tumor protein p53; p-CREB: cAMP response element-binding protein; PMC-12: Polygonum Multiflorum Thunberg Complex Composition-12; ROS: reactive oxygen species; SA-*β*-Gal: senescence-associated *β*-galactosidase; SAMP-8: senescence-accelerated mouse-prone 8; SCs: stem cells; SGZ: subgranular zone; SVZ: subventricular zone.

**Table 2:** Herb-derived products with an antisenescence role on human *ex vivo* cultured tissue-derived SCs.

Herb name	Studied cells	Treatment dose	Time exposure	Senescence inducers (dose/time)	Treatment (pre-, post-, and coinducer)	Effects on cells	Ref. number
*Andrographis paniculata*	EpSCs	1-10-30 *μ*g/mL	24 or 72 hours	Absent	/	Increase in cell proliferation through the expression of Integrin *β*1 and VEGF	[147]

*Cirsium setidens*	ASCs	100 *μ*g/mL	30 minutes	H_2_O_2_ (200 *μ*M/15-30-60-120 minutes or 8 hours)	Pre-treatment	Suppression of H_2_O_2_-induced cell damage by modulating the oxidative stress signaling pathways and by inhibiting the apoptosis-related signaling pathways	[69]

*Dhanwantharam kashaya*	WJMSCs	10 *μ*g/mL	12-24-48 hours	Absent	/	Enhancement of cell viability and proliferation; maintenance of SC features; delay in the onset of replicative senescence	[154]

*DHJST* *Ligusticum chuanxiong*	BMSCs	3-6 *μ*g/mL 1-3 *μ*g/mL	2 or 5 weeks	Absent	/	Enhancement of hMSC osteogenesis Reduction of replicative senescence	[158]

*Ginkgo biloba*	EPCs	10-25-50 mg/L	24 hours, 1 week, or 10 days	Absent	/	Increase in cell proliferation; delay in the onset of replicative senescence by increasing telomerase activity through the activation of PI3k/Akt signaling pathway	[141]

*Myrtle*	ASCs	0.5 mg/mL	12-24-48 hours	H_2_O_2_ (100 *μ*M/1 hour)	Pre-treatment	Decrease in oxidative stress and inflammation; reduction of oxidative stress-induced senescence; increase in the expression of pluripotency-related genes	[161]

*Myrtle*	ASCs	0.5 mg/mL	12-24-48 hours	H_2_O_2_ (100 *μ*M/1 hour)	Pre-treatment	Decrease in the number of SA-*β*-Gal-positive cells; decrease in the expression of cell cycle regulatory genes; upregulation of *TERT* and *c-Myc* gene expression	[162]

*Tianshengyuan-1*	HSCs	31.2-62.5 *μ*g/mL	24 hours	Absent	/	Increase in telomerase activity through the epigenetic regulation of *TERT* promoter region; decrease in the number of SA-*β*-Gal-positive cells	[164]

*Tinospora cordifolia* *Withania somnifera*	WJMSCs	10 *μ*g/mL 5 *μ*g/mL	24-48 hours	Absent	/	Enhancement of cell proliferation and viability; inhibition of cell apoptosis; delay in the onset of replicative senescence	[170]

*Undaria pinnatifida*	BMSCs	5 *μ*g/mL	24 hours	H_2_O_2_ (1 mM/1 hour)	Pre-treatment	Protection against oxidative stress in H_2_O_2_-treated cells; reduction of replicative senescence in long-term expansion cell culture by modulating ROS production; improving in differentiation potential in long-cultured cells	[172]

Herb-derived products are shown in alphabetical order. ASCs: adipose-derived stem cells; Akt: serine/threonine kinase 1; BMSCs: bone marrow-derived mesenchymal stromal cells; c-Myc: MYC protooncogene, bHLH transcription factor; DHJST: Du-Huo-Ji-Sheng-Tang; EPCs: endothelial progenitor cells; EpSCs: epidermal stem cells; H_2_O_2_: hydrogen peroxide; HSCs: hematopoietic stem cells; MSCs: multipotent mesenchymal stromal cells; PI3k: phosphatidylinositol 3-kinase; ROS: reactive oxygen species; SA-*β*-Gal: senescence-associated *β*-galactosidase; SCs: stem cells; TERT: telomerase reverse transcriptase; VEGF: vascular endothelial growth factor; WJMSCs: Wharton's jelly MSCs.

**Table 3:** Known herbal active compounds with an antisenescence role on human senescent cells.

Compound name	Studied cells	Treatment dose	Time exposure	Senescence inducers (dose/time)	Treatment (pre-, post-, and coinducer)	Effects on cells	Ref. number
Morin	KSCs	10-20-100 *μ*M	5-30-60 minutes or 24 hours	UVB exposure (30 mJ/cm^2^)	Post-treatment	Increase in cellular viability and decrease in senescence and DNA damage in UVB-treated cells; increase in the anti-inflammatory functions	[231]

Quercetin	BMSCs	100 *μ*M or 50 *μ*M in long-term treatment	1-3 days or long-term treatment	Absent	/	No senolytic effects on replicative senescent MSCs	[245]

Quercetin	WS MSCs; HGPS MSCs; physiological-aging wild-type MSCs from a 56-year subject; replicative-senescent wild-type MSCs	100 nmol/L	7 days or 30 days	Absent	/	Decrease in replicative senescence, oxidative stress, inflammation and apoptosis in WS MSCs; enhancement of osteogenic and chondrogenic differentiation in WS MSCs; attenuation of cellular senescence in HGPS MSCs and in both physiological-aging MSCs	[244]

Vanillin	KSCs	10-20-100 *μ*M	5-30-60 minutes or 24 hours	UVB exposure (30 mJ/cm^2^)	Post-treatment	Increase in cellular viability in UVB-treated cells; decrease in senescence, in DNA damage and in the production of inflammatory cytokines in UVB-treated cells	[234]

Zingerone	KSCs	10-20-100 *μ*M	60 minutes or 24 hours	UVB exposure (30 mJ/cm^2^)	Post-treatment	Enhancement of cell viability; decrease in senescence and DNA damage; decrease in the production of inflammatory cytokines	[237]

Herb compounds are shown in alphabetical order. BMSCs: bone marrow-derived mesenchymal stromal cells; HGPS: Hutchinson-Gilford progeria syndrome; MSCs: multipotent mesenchymal stromal cells; KSCs: keratinocyte stem cells; WS: Werner syndrome.

## Abbreviations

8-OHdG: 8-Hydroxy-2′-deoxyguanosine
AA: Aplastic anemia
AD: Alzheimer's disease
ADSCs: Adipose-derived stem cells
Akt: Serine/threonine kinase 1
ap2: Adipocyte protein 2
APS: Astragalus Polysaccharide
ARDs: Age-related diseases
ASP: Angelica Sinensis Polysaccharide
AT: Acori tatarinowii
ATM: ATM serine/threonine kinase
Bax: BCL2-associated X protein
BCL-2: BCL2 apoptosis regulator
BDNF: Brain-derived neurotrophic factor
BMP-2: Bone morphogenetic protein 2
BMSCs: Bone marrow-derived mesenchymal stromal cells
BrdU: Bromodeoxyuridine
CDK: Cyclin-dependent kinase
c-Fos: Fos protooncogene AP-1 transcription factor subunit
CFU: Colony-forming units
c-Myc: MYC protooncogene, bHLH transcription factor
Col1a1: Collagen type I alpha 1 chain
DG: Dentate gyrus
D-gal: D-Galactose
DHJST: Du-Huo-Ji-Sheng-Tang
DK: Dhanwantharam kashaya
DNMT1: DNA methyltransferase
EPCs: Endothelial progenitor cells
EpSCs: Epidermal stem cells
ERK: Extracellular signal regulated kinase
FAC: Ferric ammonium citrate
FZS: Fuzhisan
GBE: Ginkgo biloba extract
GFP: Green fluorescent protein
GM-CSF: Granulocyte-macrophage colony-stimulating factor
GSH: Glutathione
h: Hours
HDAC1: Histone deacetylase 1
H2AX: Histone H2AX
H_2_O_2_: Hydrogen peroxide
HPCs: Hematopoietic progenitor cells
HSCs: Hematopoietic stem cells
IL: Interleukin
Ki-67: Marker of proliferation Ki-67
KSCs: Keratinocyte stem cells
Lef: Lymphoid enhancer factor
LiCl: Lithium chloride
LSK: Lin−sca-1+c-kit+
MAP-2: Microtubule-associated protein 2
MAPKs: Mitogen-activated protein kinases
Mash1: Mammalian achaete–scute homolog 1
MDA: Malondialdehyde
MSCs: Multipotent mesenchymal stromal cells
Nanog: Nanog homeobox
NeuroD: Neuronal differentiation
NF-*κ*b: Nuclear factor kappa B subunit
Ngn1: Neurogenin 1
NPC: Neural progenitor cell
NPs: Nanoparticles
NSCs: Neural stem cells
Oct4: Octamer-binding transcription factor 4
p16 (p16Ink4a): Cyclin-dependent kinase inhibitor 2A, multiple tumor suppressor 1
p18: Cyclin-dependent kinase inhibitor 2C
p19ARF: Cyclin-dependent kinase inhibitor 2A, ARF tumor suppressor
p21: Cyclin-dependent kinase inhibitor 1A
p38MAPK: Mitogen-activated *protein* kinase *p38*
p42/44MAPK: p42/p44 mitogen-activated protein kinase
p53: Tumor protein p53
pCNA: Proliferating cell nuclear antigen
p-CREB: cAMP response element-binding protein
p-Gsk-3*β*: Glycogen synthase kinase 3 beta
PI3k: Phosphatidylinositol 3-kinase
PKM2: Pyruvate kinase
PPAR*γ*: Peroxisome proliferator-activated receptor gamma
PMC-12: Polygonum Multiflorum Thunberg Complex Composition-12
ROS: Reactive oxygen species
RUNX2: RUNX family transcription factor 2
SA-*β*-Gal: Senescence-associated *β*-galactosidase
SAMP: Senescence-accelerated mouse-prone
SASP: Senescence-associated secretory phenotype
SCs: Stem cells
SCF: Stem cell factor
SGZ: Subgranular zone
SIPS: Stress-induced premature senescence
SIRTs: Sirtuins
SOD: Superoxide dismutase
Sox2: SRY-box transcription factor 2
SVZ: Subventricular zone
T-AOC: Total antioxidant
t-BHP: tert-Butyl hydrogen peroxide
Tcf: T-cell factor
TERT: Telomerase reverse transcriptase
TGF: Transforming growth factor
TMP: Tetramethylpyrazine
TNF: Tumor necrosis factor
TSY-1: Tianshengyuan-1
YKS: Yokukansan
VEGF: Vascular endothelial growth factor
WJMSCs: Wharton's jelly mesenchymal stromal cells.

## Supplementary Materials

Supplementary Materials Table S1: known herbal active compounds with an antisenescence role on animal senescent cells. Herbal compounds are shown in alphabetical order.
