# Supplementary material for: Herb-Derived Products: Natural Tools to Delay and Counteract Stem Cell Senescence
Source: Stem Cells Int. 2020 Oct 8;2020:8827038. doi: 10.1155/2020/8827038 (PMC7568162; doi:10.1155/2020/8827038)
Supplement: Supplementary Materials — Table S1: known herbal active compounds with an antisenescence role on animal senescent cells. Herbal compounds are shown in alphabetical order. [file 8827038.f1.docx]

| **Compound**  **name** | **Studied cells** | **Animal source** | **Treatment dose** | **Time exposure** | **Senescence inducers (dose of exposure)** | **Treatment (pre-, post-, co-inducer)** | **Effects on recovered cells** | **Effects on animals or cells in animals after treatment** | **Ref. number** |
| --- | --- | --- | --- | --- | --- | --- | --- | --- | --- |
| Allicin | HSCs recovered from animals after treatment | C57BL/6 mice | 30 μg/kg/day (animals) | 8 weeks (animals) | lead acetate (tap water with 0.2%) for 8 weeks  (animals) | co-treatment | improved colony-forming ability of LSK population containing HSCs;  attenuated cellular ROS production and DNA damage;  alleviated cell senescence by up-regulating PKM2 | ameliorated SASP features by reducing IL-6 and TNF-α levels in peripheral blood;  reversal of the imbalance in the differential population of myeloid and lymphoid cells in the bone marrow;  ameliorated self-renewal function of cells | [208] |
| Angelica sinensis polysaccharide | hippocampus resident NSCs after animal treatment  NSCs recovered from animals | Nestin-GFP transgenic mice (2 months old) | 140 mg/kg/day (animals)  100 μg/mL (cells) | for 28 days from day 15 of D-gal exposure (animals)  for 24 hours (cells) | D-glucose (200 mg/kg/day) for 42 days (animals)  D-glucose (20 mg/mL/day) for 48 hours (cells) | post-treatment  post-treatment | increased cell proliferation in a dose-dependent manner;  increased cell number;  increased activity of SOD and T-AOC;  reduced level of MDA and ROS;  reduced inflammatory cytokines ( IL-1 β, IL-6, TNF-α);  down-regulated expression of senescence associated genes *p53*, *p21* | reduced cognitive impairment;  increased activity of Na+-K+ -ATPase in hippocampus;  decreased percentage of senescent neurospheres in hippocampus | [196] |
| Angelica sinensis polysaccharide | HSC/HPCs recovered from animals after treatment | C57BL/6J mice  (6-8 weeks old) | 200 mg/kg/day (animals) | for 35 days from day 8 of D-gal exposure (animals) | D-glucose (120 mg/kg/day) for 42 days (animals) | post-treatment | reduced SA-β-Gal + cells;  enhanced capacity to form colony unit;  declined levels of ROS;  reduced expression of 𝛾-H2A.X;  decreased expressions of p16, p19 and p21;  inhibited excessive activation of Wnt/β-catenin signaling | / | [194] |
| Angelica sinensis polysaccharide | HSCs recovered from animals after treatment | C57BL/6J mice | unknown | unknown | X-ray radiation (3.0 Gy/8 F) (animals) | co-treatment | inhibited ratio of cells in G1 stage and the number of SA-β-Gal + cells;  down-regulated expression of p53;  increased length of telomere and vitality of telomerase | / | [193] |
| Angelica sinensis polysaccharide | HSCs recovered from animals after treatment | C57BL/6J mice | unknown | unknown | X-ray radiation (3.0 Gy/8 F) (animals) | co-treatment | inhibited ratio of cells in G1 stage and the number of SA-beta-Gal + cells;  down-regulated expression of p16 mRNA;  reduced production of ROS and enhanced capability of T-AOC | / | [192] |
| Astragalus polysaccharide | BMSCs recovered from animals | C57BL/6J mice | from 30 to 100 μg/mL (cells) | 24 hours (cells) | FAC (200 μM) for 24 hours (cells) | co-treatment | enhanced proliferation and viability;  increased expression of *Nanog*, *Sox2* and *Oct4*;  decreased intracellular and mitochondrial ROS level;  attenuated apoptosis modulating Bax/Bcl-2 expression;  reduced percentage of SA-β-Gal + cells | / | [79] |
| Astragali Radix combined with Angelicae Sinensis Radix 1:1 | HSCs recovered from animals | C57BL/6J mice | unknown | unknown | t-BHP (unknown) (cells) | unknown | inhibited cell senescence;  promoted cell proliferation;  up-regulated expression of cell cycle positive regulators and down-regulated expression of negative ones | / | [201] |
| Curcumin | ASCs recovered from animals | Sprague-  Dawley rats | 1 and 5 µM (cells) | 48 hours (cells) | absent | / | increased cell proliferation;  decreased number of senescent cells;  increased expression of *TERT1* |  | [218] |
| Curcumin | BMSCs recovered from animals | Sprague-  Dawley rats  (8-10 weeks old) | 2 μM and 10 μM (cells) | 3, 6, 9 and 12 days (cells) | absent | / | decreased cell proliferation;  enhanced cell viability;  decreased *p53* and *p16* gene expression;  reduced percentage of SA-β-Gal + cells |  | [217] |
| Ginsenoside Rg1 | NSCs recovered from animals | Nestin-GFP transgenic newborn rats | 20 μg/mL (cells) | 1 day (cells) | LiCl (20 mmol/L) for 1 day  (cells) | post-treatment | enhanced number of neurospheres;  increased number of proliferative cells;  reduced percentage of senile neurospheres;  increased presence of cytoplasmic β-catenin and decreased expression of nuclear catenin, Tcf, Lef, p-Gsk-3β, and c-myc | / | [85] |
| Ginsenoside Rg1 | BMSCs recovered from animals after treatment  BMSCs recovered from animals | Sprague-Dawley rats (6–8 weeks old ) | 20 mg/kg/day (animals)  final concentration 8 μg/mL (cells) | for 28 days from day 15 of D-gal injection (animals)  48 hours (cells) | D-gal (120 mg/kg/day) for 42 days (animals)  D-gal (final concentration 30 mg/mL) for 48 hours (cells) | post-treatment  co-treatment | reduced SA-β-Gal staining;  decreased number of apoptotic bodies;  decreased ratio of G0/G1 phase; increased ratio of S phase;  enhanced levels of GM-CSF, SCF, IL-6 and IL-1β;  decreased senescence-associated protein expression: p16, p21 and p53 | / | [174] |
| Ginsenoside Rg1 | HSCs recovered from animals after treatment | Sprague-Dawley rats (8–10 weeks old) | up to 40 mg/kg/day (animals) | 4 weeks (in animals) | lead acetate (tap water with 0.2%) for 12 weeks (animals) | post-treatment | reduced SA-β-Gal + cells in a dose-dependent manner;  reduced expression of p53 and of γ-H2AX;  reduced level of TNF-α, IL-6 in a relative dose-dependent manner;  enhanced CFU ability | restored hematological parameters to a nearly physiological level | [83] |
| Ginsenoside Rg1 | HSCs recovered from animals after treatment | C57BL/6 mice (6–8 weeks old) | 20 mg/kg/day (animals) | for 35 days from day 8 of D-gal injection (animals) | D-gal (120 mg/kg/day) for 42 days  (animals) | post-treatment | reduced SA-β-Gal staining;  enhanced CFU ability;  adjusted oxidative stress indices like ROS, T-AOC, SOD, GSH and MDA;  down-regulated advanced glycation end products;  down-regulated H2A.X and 8-OHdG | / | [74] |
| Ginsenoside Rg1 | BMSCs recovered from animals after treatment | Sprague-Dawley rats (3 months old) | 20 mg/kg/day (animals) | for 28 days from day 15 of D-gal injection (animals) | D-gal (120 mg/kg)/day for 42 days (animals) | post-treatment | decreased levels of SA-β-Gal+ cell %;  increased percentages of cells in S phase;  increased cell proliferation;  decreased ROS and MDA presence; increased SOD activity;  decreased expression of inflammatory markers (IL-2, IL-6 and TNF-α ) and increased SCF expression;  decreased senescence-associated protein expression: p16 , p21 and p53 | / | [181] |
| Ginsenoside Rg1 | Sca‑1+ HSC/HPCs recovered from animals  Sca‑1+ HSC/HPCs recovered and transplanted in animals after their Rg1 treatment | C57BL/6 mice (6‑8 weeks old) | 10 μmol/L (cells) | 12 hours (cells) | t‑BHP (100 μmol/L) for 6 hours (cells)  X-ray (8.5 Gy) for 8.5 min (animals) | post-treatment  post-treatment | decreased SA-β-Gal staining;  compensated ability to form colonies;  decreased expression of p16;  alleviated cell cycle arrest | reconstituted telomerase activity;  activation of genes involved in p16‑Rb signaling pathways | [182] |
| Ginsenoside Rg1 | Sca-1+ HSC/HPCs recovered from animals | C57BL/6 mice (6‑8 weeks old) | 10 μmol/L (cells) | 6 hours (cells) | t‑BHP (100 μmol/L) for 6 hours (cells) | pre- and post-treatment | pre-treatment had more remarkable effects:  increased mRNA and protein expression of SIRT6;  down-regulated mRNA and protein expression of NF-κB;  decreased percentage of  SA-β-Gal + cells;  decreased cells in G1 phase;  increased number of CFU |  | [55] |
| Ginsenoside Rg1 | NSCs/NPCs recovered from animals after treatment | Sprague-Dawley rats (3 months old) | 20 mg/kg/day (animals) | for 28 days from day 15 of D-gal injection (animals) | D-gal (20 mg/kg/day) for 42 days (animals) | post-treatment | increased hippocampal cell proliferation;  enhanced activity of the antioxidant enzymes;  decreased levels of IL-1β, IL-6 and TNF-α;  increased DNA telomere lengths;  down-regulated expression of *p53*, *p21* and *p19* | attenuated changes in the hippocampus, including cognitive capacity, senescence-related markers, hippocampal neurogenesis | [177] |
| Ginsenoside Rg1 | HSC/HPCs recovered from animals after treatment | C57BL/6 mice (6–8 weeks old) | 20 mg/kg/day (animals) | 7 days (animals) | X-ray (6.5 Gy)  (animals) | pre-treatment | decreased SA-β-Gal staining;  reduced G1 phase arrest;  increased SOD activity;  decreased MDA contents and reduced DNA damage;  decreased expression of p16 and p21 | / | [87] |
| Ginsenoside Rg1 | HSCs recovered from animals | C57BL/6 mice (6-8-weeks old) | 10 μM (cells) | 6 hours (cells) | t-BHP (100 μM) for 6 hours (cells) | pre- and post-treatment | pre-treatment had more remarkable effects:  reduced SA-β-Gal + cells;  prevented decrease in colony number;  decreased expression of p16, p21 and cyclinD1 | / | [183] |
| Icariin | hippocampus resident NSCs after animal treatment | Sprague-Dawley rats (18 months-old) | 0.02 g/kg/day (animals) | 3 months  (animals) | absent | / | / | stimulated animal spatial learning and memory;  increased number of BrdU and GFAP double-labeled cells | [209] |
| Quercetin | SCs recovered from animals after treatment | progeroid Ercc1+/D mice or 24-months-old C57Bl/6 mice | 50 mg/kg/day (animals) | 10-12 weeks (animals) | absent | / | reduced number of SA-β-Gal + cells | extended health span, delaying age-related symptoms and pathology; reduced senescence markers in several tissues;  reduced expression of p16 mRNA in fat and liver of 24-months-old mice | [242] |
| Sesamin | SCs resident in intestine after  animal tretment | Drosophila senescence-accelerated model | 2 mg/mL (animals) | every 3 days until 30 days (animals) | absent | / | suppressed DNA damage accumulation;  suppressed cell hyperproliferation | extended lifespan;  suppressed age-dependent loss of locomotor activity;  inhibited accumulation of ROS;  reduced loss of dopaminergic neurons in adult brains | [254] |
| Tetramethylpyrazine | BMSCs recovered from animals | Sprague–Dawley rats (3–4 weeks old) | (0, 20, 30, 40, 50, 60, 70 and 80 mg/L) (cells) | for 1, 2, 4, 6, and 8 days  (cells) | absent | / | increased cell viability;  decreased SA-β-Gal + cells;  suppressed NF-κB signaling with reduced expression of inflammatory factors TNF-α and IL-1β;  promoted cell proliferation by regulating the cell cycle;  facilitated neuronal differentiation;  promoted expression of the neuronal markers Ngn1, NeuroD and Mash1 | / | [228] |

8-OHdG: 8-hydroxy-2′-deoxyguanosine; ASCs: adipose‑derived stem cells; Bax: BCL2-associated X protein; BCL-2: BCL2 apoptosis regulator; BMSCs: bone marrow-derived mesenchymal stromal cells ; BrdU: bromodeoxyuridine; CFU: colony forming units; c-Myc: MYC proto-oncogene, bHLH transcription factor; DG: dentate gyrus; D-gal: D-galactose; FAC: ferric ammonium citrate; GFP: green fluorescent protein; GM-CSF: granulocyte-macrophage colony-stimulating factor; Gsk-3β: glycogen synthase kinase 3 beta; GSH: glutathione; H2AX: histone H2AX; H_2_O_2_: hydrogen peroxide; HPCs: hematopoietic progenitor cells; HSCs: hematopoietic stem cells; IL: interleukin; Ki-67: marker of proliferation Ki-67; Lef: lymphoid enhancer factor; LSK: lin−sca-1+c-kit+; MAP-2: microtubule-associated protein 2; Mash1: mammalian achaete–scute homolog 1; MDA: malondialdehyde; Nanog: Nanog homeobox; NeuroD: neuronal differentiation; NF-Kb: nuclear factor kappa B subunit; Ngn1: neurogenin 1; NPC: neural progenitor cell; NSCs: neural stem cells; Oct4: octamer-binding transcription factor 4; p16: cyclin dependent kinase inhibitor 2A, multiple tumor suppressor 1; p19: cyclin dependent kinase inhibitor 2A, ARF tumor suppressor; p21: cyclin-dependent kinase inhibitor 1A; p53: tumor protein p53; PKM2: pyruvate kinase; ROS: reactive oxygen species; SA-β-Gal: senescence-associated β-galactosidase; SASP: senescence-associated secretory phenotype; SCs: stem cells; SCF: stem cell factor; SIRT: sirtuin; SOD: superoxide dismutase; Sox2: SRY-box transcription factor 2; T-AOC: total anti-oxidant; t-BHP: tert-butyl hydrogen peroxide; Tcf: T-cell factor; TERT: telomerase reverse transcriptase; TGF: transforming growth factor; TNF: tumor necrosis factor
